# Supplementary material for: Causal inference on microbiome-metabolome relations in observational host-microbiome data via in silico in vivo association pattern analyses
Source: Cell Rep Methods. 2023 Oct 16;3(10):100615. doi: 10.1016/j.crmeth.2023.100615 (PMC10626217; doi:10.1016/j.crmeth.2023.100615)
Supplement: Document S1. Figure S1 and Note S1 [file mmc1.pdf]

Cell Reports Methods, Volume *3*

Supplemental information

Causal inference on microbiome-metabolome relations in observational host-microbiome data via *in silico in vivo* association pattern analyses

Johannes Hertel, Almut Heinken, Daniel Fässler, and Ines Thiele

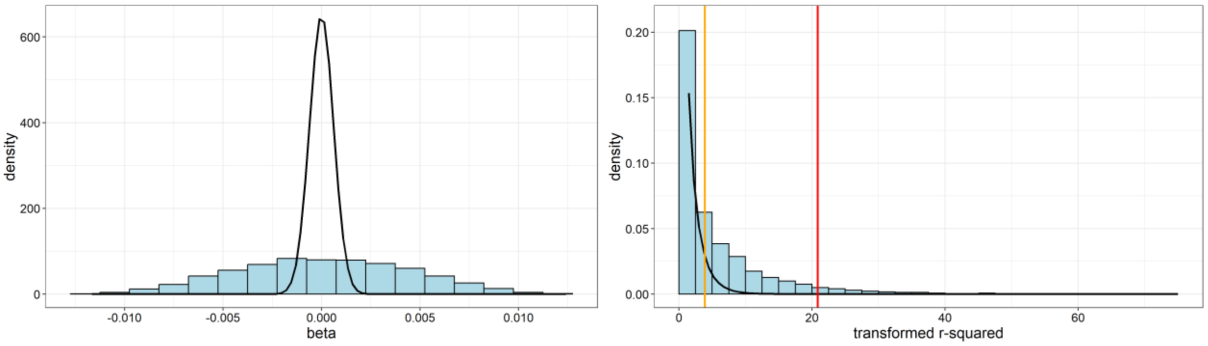

Figure S1. Histogram of estimated regression coefficients (beta) characterising *in silico in vivo* association pattern from 5,000 permutations, related to STAR Methods. Histogram of estimated regression coefficients (beta) characterising *in silico in vivo* association pattern from 5,000 permutations of butyrate net secretion fluxes and the expected density of the normal distribution (left panel). On the right panel the distribution of the corresponding transformed R^2 values, density function of the $\mathrm{F}(1{,}146)$ distribution (black), the 5% quantile (orange line) and the empirical 5% quantile of the transformed R^2 (red line) are shown. Empirical distributions indicate that nominal p-values will be too liberal.

Note S1: Formalisation of metabolic host-microbiome systems via structural equation modelling, related to Figure 2

As noted in the main body of the manuscript, we define:

a) $Microbiome\ community\ composition\ \boldsymbol{a} := (a_1, a_{2,}, \dots, a_L) \in [0,1]^L\ with\ \sum_{l=1}^{L} a_l = 1$
b) $Host\ characterisation\ \boldsymbol{h} := (h_1, h_{2,}, \dots, h_K) \in \mathbb{R}^K$
c) $Community\ net\ secretion\ \boldsymbol{f} := (f_1, f_{2,}, \dots, f_J) \in \mathbb{R}^J\ of\ J\ metabolite\ fluxes$
d) $Host\ metabolome\ \boldsymbol{c} := (c_1, c_{2,}, \dots, c_J) \in \mathbb{R}^J\ of\ J\ metabolite\ concentrations$

We will call the vector $(\boldsymbol{a}, \boldsymbol{h}, \boldsymbol{f}, \boldsymbol{c})$ metabolic microbiome-host-system, dealing with *N* independent and identically distributed (IID) realisations of the vector $(\boldsymbol{a}, \boldsymbol{h}, \boldsymbol{c})_{\boldsymbol{1}}, (\boldsymbol{a}, \boldsymbol{h}, \boldsymbol{c})_{\boldsymbol{2}}, \dots, (\boldsymbol{a}, \boldsymbol{h}, \boldsymbol{c})_{\boldsymbol{N}}$, with $\boldsymbol{f_n}$ being approximated by a deterministic known function based on microbiome composition $\boldsymbol{a_n}$, the genome-scale reconstructions of the microbes detected in $\boldsymbol{a_n}$ and constant diet constraints across the whole population of metabolic microbiome-host-systems. Notably, we do not need to apply distributional assumptions other than the metabolic microbiome-host-systems being IID. We will generally assume that (a, h, f, c) are nontrivial with finite variances. The whole methodology is therefore nonparametric.

We assume that the interrelations can be reasonably approximated by the following structural linear equation system:

(1) $a_l = u_l + \sum_{k=1}^{K} b_{ah_{lk}} h_k$, leading to $\boldsymbol{a} = \mathbf{B_{ah}} \boldsymbol{h} + \boldsymbol{u}\ with\ \mathbf{B_{ah}} \in MAT(L, K)\ and\ \boldsymbol{u} = (u_1, u_{2,}, \dots, u_L)$,

(2) $f_j = \sum_{l=1}^{L} b_{fa_{jl}} a_l$, leading to $\boldsymbol{f} = \mathbf{B_{fa}} \boldsymbol{a}\ with\ \mathbf{B_{fa}} \in MAT(J, L)$,

(3) $c_j = \sum_{i=1}^{J} b_{cf_{ji}} f_i + \sum_{k=1}^{K} b_{ch_{jk}} h_k + \varepsilon_j, leading\ to\ \boldsymbol{c} = \mathbf{B_{cf}} \boldsymbol{f} + \mathbf{B_{ch}} \boldsymbol{h} + \boldsymbol{\varepsilon}\ with\ \mathbf{B_{cf}} \in MAT(J, J),\ \mathbf{B_{ch}} \in MAT(J, K),\ \boldsymbol{\varepsilon} = (\varepsilon_1, \varepsilon_{2,}, \dots, \varepsilon_J)$.

As described in the main text, we assume:
i) $\boldsymbol{u} \perp\!\!\!\perp \boldsymbol{h}, (stochastic\ independence\ between\ \boldsymbol{u}\ and\ \boldsymbol{h})$
ii) $\boldsymbol{\varepsilon} \perp\!\!\!\perp \boldsymbol{h}, \boldsymbol{f}\ (stochastic\ independence\ between\ \boldsymbol{\varepsilon}\ and\ \boldsymbol{h}, \boldsymbol{f})$
iii) $\mathbf{B_{fa}} \mathbf{B_{ah}} = \mathbf{0}\ (approximate\ orthogonality\ of\ \mathbf{B_{ah}}\ and\ \mathbf{B_{fa}})$

In this supplementary note, we will derive three attributes of the above constructed system of structural equations.

We define furthermore as in the main document:
(4) $\hat{b}_{fa_{jl}} = \frac{COV(f_j, a_l)}{VAR(a_l)}$, $\hat{\boldsymbol{b}}_{\boldsymbol{fa}_{j.}} = (\hat{b}_{fa_{j1}}, \hat{b}_{fa_{j2}}, \dots, \hat{b}_{fa_{jL}})$ *(in silico association statistics)*
which is the vector of regression coefficients of the regression prediction score:
(5) $\hat{f}_j = \hat{b}_{fa_{jl}} a_l + b_{fa_0}$.
Second, we define
(6) $\hat{b}_{ca_{jl}} = \frac{COV(c_j, a_l)}{VAR(a_l)}$, $\hat{\boldsymbol{b}}_{\boldsymbol{ca}_{j.}} = (\hat{b}_{ca_{j1}}, \hat{b}_{ca_{j2}}, \dots, \hat{b}_{ca_{jL}})$ *(in vivo association statistics)*
which is vector of the regression coefficients of the regressions prediction score:
(7) $\hat{c}_j = \hat{b}_{ca_{jl}} a_l + b_{ca_0}$.

Attribute 1:

The covariance $COV\left(\boldsymbol{b}_{\boldsymbol{fa}_{j.}}, \boldsymbol{b}_{\boldsymbol{ah}_{.k}}\right)$ between the vectors of model parameters $\boldsymbol{b}_{\boldsymbol{fa}_{j.}} = (b_{fa_{j1}}, b_{fa_{j2}}, \dots, b_{fa_{jL}})$ and $\boldsymbol{b}_{\boldsymbol{ah}_{.k}} = (b_{ah_{1k}}, b_{ah_{2k}}, \dots, b_{ah_{Lk}})$ is zero if $Var(\sum_{k=1}^{K} b_k h_k) \neq 0$ for all nontrivial coefficient vectors with $\|(b_1, b_2, \dots, b_K)\| \neq 0$.

Proof:

From iii) and ii), it follows that $\sum_{l=1}^{L} b_{ah_{lk}} = 0$ for all $k \in \{1, 2, \dots, K\}$, since $\sum_{l=1}^{L} a_l = 1$
(8) $1 = \sum_{l=1}^{L} a_l = \sum_{l=1}^{L} (u_l + \sum_{k=1}^{K} b_{ah_{lk}} h_k) = \sum_{l=1}^{L} u_l + \sum_{l=1}^{L} \sum_{k=1}^{K} b_{ah_{lk}} h_k = \sum_{l=1}^{L} u_l + \sum_{k=1}^{K} h_k \sum_{l=1}^{L} b_{ah_{lk}}$.

As $\boldsymbol{u} \perp\!\!\!\perp \boldsymbol{h}$:

(9) $0 = COV\left(\sum_{l=1}^{L} u_l, \sum_{j=1}^{J} h_j \sum_{l=1}^{L} b_{ah_{lj}}\right) = COV\left(\sum_{l=1}^{L} u_l, \sum_{j=1}^{J} h_j \sum_{l=1}^{L} b_{ah_{lj}}\right) = COV\left(1 - \sum_{l=1}^{L}\sum_{k=1}^{K} b_{ah_{lk}} h_k, \sum_{j=1}^{J} h_j \sum_{l=1}^{L} b_{ah_{lj}}\right) = -COV\left(\sum_{k=1}^{K} h_k \sum_{l=1}^{L} b_{ah_{lk}}, \sum_{j=1}^{J} h_j \sum_{l=1}^{L} b_{ah_{lj}}\right) = -Var\left(\sum_{k=1}^{K} h_k \sum_{l=1}^{L} b_{ah_{lk}}\right).$

Thus, $\sum_{l=1}^{L} b_{ah_{lk}} = 0$ for all $k \in \{1,2,\ldots,K\}$. Rewriting $COV\left(\boldsymbol{b_{fa_{j\cdot}}}, \boldsymbol{b_{ah_{\cdot k}}}\right)$ as difference between arithmetic means, we get:

(10) $COV\left(\boldsymbol{b_{fa_{j\cdot}}}, \boldsymbol{b_{ah_{\cdot k}}}\right) = Mean\left(\boldsymbol{b_{fa_{j\cdot}}} \boldsymbol{b_{ah_{\cdot k}}}\right) - Mean\left(\boldsymbol{b_{ah_{\cdot k}}}\right) Mean\left(\boldsymbol{b_{fa_{j\cdot}}}\right) = \frac{1}{L}\sum_{i=1}^{L} b_{fa_{ji}} b_{ah_{ik}} - \frac{1}{L}\sum_{i=1}^{L} b_{fa_{ji}} \frac{1}{L}\sum_{i=1}^{L} b_{ah_{ik}} = \frac{1}{L}\sum_{i=1}^{L} b_{fa_{ji}} b_{ah_{ik}} = 0, since\ \mathbf{B_{fa}B_{ah}} = \mathbf{0}.$

□

Attribute 2:

The estimate $\hat{b}_{fa_{jl}}$ is a function of $\boldsymbol{u}$ and $\mathbf{B_{fa}}$ with $\hat{b}_{fa_{jl}} = \frac{COV\left(\sum_{i=1}^{L} b_{fa_{ji}} u_i, u_l\right)}{Var(a_l)}$.

Proof:

We get utilising that $\mathbf{B_{fa}B_{ah}} = \mathbf{0}$ and that $\boldsymbol{u} \perp\!\!\!\perp \boldsymbol{h}$:

(8) $VAR(a_l)\hat{b}_{fa_{jl}} = COV(f_j, a_l) = COV\left(\sum_{i=1}^{L} b_{fa_{ji}} a_i, a_l\right) = COV\left(\sum_{i=1}^{L} b_{fa_{ji}}\left(u_i + \sum_{k=1}^{K} b_{ah_{ik}} h_k\right), a_l\right) = COV\left(\sum_{i=1}^{L} b_{fa_{ji}} u_i + \sum_{i=1}^{L} b_{fa_{ji}} \sum_{k=1}^{K} b_{ah_{ik}} h_k, a_l\right) = COV\left(\sum_{i=1}^{L} b_{fa_{ji}} u_i, a_l\right) + COV\left(\sum_{i=1}^{L} b_{fa_{ji}} \sum_{k=1}^{K} b_{ah_{ik}} h_k, a_l\right) = COV\left(\sum_{i=1}^{L} b_{fa_{ji}} u_i, a_l\right) + COV\left(\sum_{k=1}^{K} h_k \sum_{i=1}^{L} b_{fa_{ji}} b_{ah_{ik}}, a_l\right) = COV\left(\sum_{i=1}^{L} b_{fa_{ji}} u_i, a_l\right) = COV\left(\sum_{i=1}^{L} b_{fa_{ji}} u_i, u_l + \sum_{k=1}^{K} b_{ah_{ik}} h_k\right) = COV\left(\sum_{i=1}^{L} b_{fa_{ji}} u_i, u_l\right).$

Thus, the estimate $VAR(a_l)\hat{b}_{fa_{jl}}$ is only dependent on $\boldsymbol{u}$ (microbiome intrinsic) and $\mathbf{B_{fa}}$.

□

Attribute 3:
The estimate $\hat{b}_{ca_{jl}}$ can be written as $\hat{b}_{ca_{jl}} = [b_{cf_{jj}}\hat{b}_{fa_{jl}} + COV(\sum_{k=1}^{K} b_{ch_{jk}} h_k, \sum_{t=1}^{K} b_{ah_{lt}} h_t)]\frac{1}{VAR(a_l)}$.

Proof:

Using the structural equations (1)-(3), we derive:

(9) $VAR(a_l)\hat{b}_{ca_{jl}} = COV(c_j, a_l) = COV\left(b_{cf_{jj}} f_j + \sum_{k=1}^{K} b_{ch_{jk}} h_k + \varepsilon_j, a_l\right) = b_{cf_{jj}} COV(f_j, a_l) + COV(\sum_{k=1}^{K} b_{ch_{jk}} h_k, a_l) = b_{cf_{jj}}\hat{b}_{fa_{lj}} + COV(\sum_{k=1}^{K} b_{ch_{jk}} h_k, u_l + \sum_{t=1}^{T} b_{ah_{lt}} h_t) = b_{cf_{jj}}\hat{b}_{fa_{lj}} + COV(\sum_{k=1}^{K} b_{ch_{jk}} h_k, \sum_{t=1}^{T} b_{ah_{lt}} h_t).$

□
