# Supplementary material for: Causal inference on microbiome-metabolome relations in observational host-microbiome data via in silico in vivo association pattern analyses
Source: Cell Rep Methods. 2023 Oct 16;3(10):100615. doi: 10.1016/j.crmeth.2023.100615 (PMC10626217; doi:10.1016/j.crmeth.2023.100615)
Supplement: Document S2. Article plus supplemental information [file mmc7.pdf]

Cell Reports
Methods

Article

Causal inference on microbiome-metabolome relations in observational host-microbiome data via *in silico in vivo* association pattern analyses

Graphical abstract

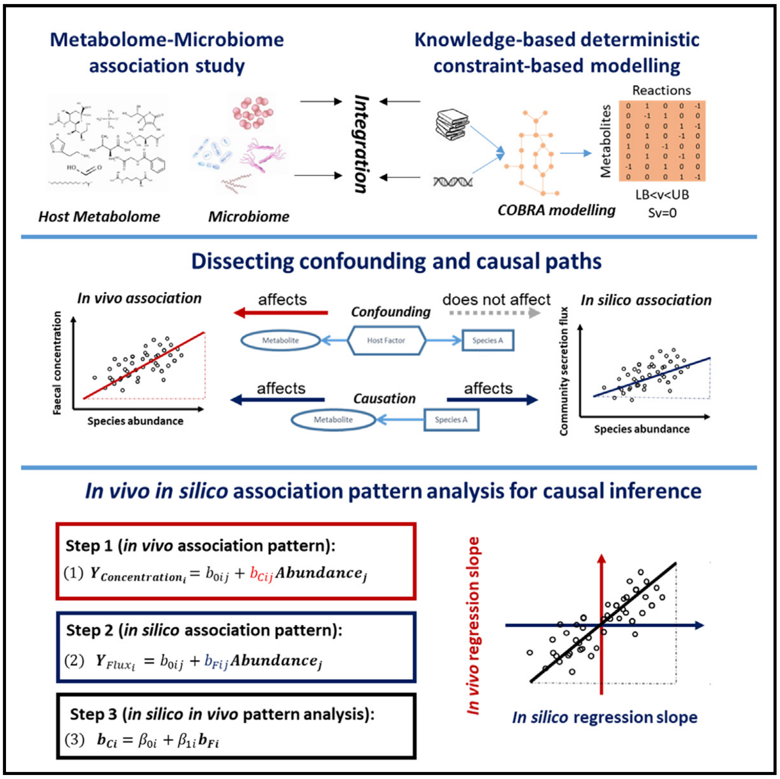

Highlights

- We conceptualize confounding and causal paths in microbiome-metabolome data
- We integrate knowledge-based and hypothesis-free modeling for causal inference
- Constraint-based models predict size and sign of metabolite-microbe associations
- We demonstrate causal microbiome-metabolite relations for 26 fecal metabolites

Authors

Johannes Hertel, Almut Heinken, Daniel Fässler, Ines Thiele

Correspondence

ines.thiele@universityofgalway.ie

In brief

Dissecting confounding from causation is key to the interpretation of statistical associations in metabolome-microbiome multi-omics data. Hertel et al. integrate deterministic constraint-based microbiome community modeling with microbiome metabolome association studies to generate a paradigm that allows for the identification of causal relations between microbiome composition and the host's metabolome.

Hertel et al., 2023, Cell Reports Methods *3*, 100615
October 23, 2023 © 2023 The Author(s).
https://doi.org/10.1016/j.crmeth.2023.100615

CellPress

Cell Reports Methods

CellPress
OPEN ACCESS

Article

Causal inference on microbiome-metabolome relations in observational host-microbiome data via *in silico in vivo* association pattern analyses

Johannes Hertel,[1,2] Almut Heinken,[1,3] Daniel Fässler,[2] and Ines Thiele[1,4,5,6,7,*]

[1]School of Medicine, University of Galway, Galway, Ireland
[2]Department of Psychiatry and Psychotherapy, University Medicine Greifswald, Greifswald, Germany
[3]UMRS Inserm 1256 NGERE (Nutrition-Genetics-Environmental Risks), Institute of Medical Research (Pôle BMS) - University of Lorraine, Vandoeuvre-les-Nancy, France
[4]Discipline of Microbiology, University of Galway, Galway, Ireland
[5]APC Microbiome Ireland, University College Cork, Cork, Ireland
[6]Ryan Institute, University of Galway, Galway, Ireland
[7]Lead contact
*Correspondence: ines.thiele@universityofgalway.ie
https://doi.org/10.1016/j.crmeth.2023.100615

MOTIVATION Causal inference on microbiome-host relations in the domain of metabolism using observational data is often intractable due to the multivariate nature of both omics classes and the effects of unmeasured confounders. However, given the emerging importance of the microbiome as an interventional target, understanding the causal effects of the microbiome on the host's metabolome becomes crucial to designing and testing the efficacy of pre-biotic and pro-biotic interventions. To aid the task of causal inference on observational microbiome-metabolome data, we propose integrating knowledge-based deterministic constraint-based modeling of microbiome communities with statistical multi-omics analysis. Analyzing confounding and causal paths, we demonstrate that this integration allows for causal inference on microbiome-metabolite relations, even in the presence of unmeasured confounders.

SUMMARY

Understanding the effects of the microbiome on the host's metabolism is core to enlightening the role of the microbiome in health and disease. Herein, we develop the paradigm of *in silico in vivo* association pattern analyses, combining microbiome metabolome association studies with *in silico* constraint-based community modeling. Via theoretical dissection of confounding and causal paths, we show that *in silico in vivo* association pattern analyses allow for causal inference on microbiome-metabolome relations in observational data. We justify the corresponding theoretical criterion by structural equation modeling of host-microbiome systems, integrating deterministic microbiome community modeling into population statistics approaches. We show the feasibility of our approach on a published multi-omics dataset (n = 347), demonstrating causal microbiome-metabolite relations for 26 out of 54 fecal metabolites. In summary, we generate a promising approach for causal inference in metabolic host-microbiome interactions by integrating hypothesis-free screening association studies with knowledge-based *in silico* modeling.

INTRODUCTION

The determination of the microbiome's metabolic functions is a key challenge in understanding the contribution of the gut microbiome to health and disease.[1–3] As metabolic functions are shared across phylogenetic classes,[4] differences in composition do not necessarily translate into differences in metabolic output. Therefore, analyses of the microbiome composition alone cannot give conclusive insights into the collective metabolic output of a microbial community. Researchers have repeatedly tried to shine a light on metabolic functions of microbes via integrating microbial abundance data with metabolome data in statistical association studies.[5–8] In particular, fecal metabolomics, being closest to a direct functional readout, has been used in statistical screenings for associations.[7–9] However, statistical screenings can easily result in false positives,[10] and recent modeling efforts have shown that microbe-metabolite associations may be the result of confounding due to the multivariate

This is an open access article under the CC BY license (http://creativecommons.org/licenses/by/4.0/).

nature of both types of omics datasets.[11] Moreover, from the viewpoint of causal statistics, extracting causal models from observational data requires full information on all relevant confounding variables.[12] However, measuring and conceptualizing all relevant confounders poses conceptual and practical problems for concrete microbiome-metabolome study, partly due to limited knowledge on relevant confounding factors.[13] Hence, results of statistical hypothesis-free screening approaches are often difficult to interpret and embed into the knowledge already gathered about microbial biology.[13,14]

Integrating the genetic content of the microbial community with knowledge of microbial biology while respecting basic laws of nature, such as conservation of mass and charge, constraint-based reconstruction and analysis (COBRA),[15] allows a fine-graded mapping of metabolic functions of microbial communities.[16] Thus, COBRA is suited to complement microbiome-metabolome association studies by delivering biological context in a quantitative way.[17] COBRA microbial community models offer quantifications of the feasible range of metabolic fluxes given a diet, allowing the calculation of, for instance, the secretion potential of a quantified microbial community into a simulated fecal compartment *in silico*.[16] In contrast to species metabolome association analysis, COBRA community modeling enables the direct deterministic calculation of species contributions to the output of the whole community.[17] As such, COBRA modeling results are not impacted by confounding caused by physiological and behavioral attributes of the host in the same way as statistical associations. COBRA microbial community modeling has already been applied to investigate metabolic functions in, e.g., Parkinson's disease[18,19] and inflammatory bowel disease,[17] showing promise in investigating microbiome-host interactions in health and disease.

Here, we develop the theoretical frameworks to combine COBRA community modeling with microbiome-metabolome association studies, outlining the causal and confounding paths effective in species-metabolite associations *in vivo* and *in silico*. Building on these theoretical considerations rooted in causal inference theory, we develop a methodological paradigm, which we call "*in silico in vivo* association pattern analyses," allowing for causal inference on microbiome-metabolite relations in theory. Using published metagenomic data in conjunction with fecal metabolome data from Yachida et al.,[7] we then demonstrate that microbial community modeling systematically predicts the *in vivo* statistical associations pattern between microbiome measurements and fecal metabolome measurements, justifying the proposed methodology. Our work highlights how metabolomics and metagenomics in combination with COBRA microbial community modeling can be utilized to improve mechanistic understanding, generate hypotheses, and identify and validate biomarkers for metabolic functions in human health and disease.

RESULTS

Theoretical frameworks

The challenge presented by *in vivo* species-metabolite association studies, especially with observational data, is to disentangle the various sources of correlation. To this end, we first classify the various sources of correlation between species and fecal metabolite concentrations *in vivo*. In a second step, we examine how these correlation sources influence *in silico* species-flux associations derived from COBRA microbial community models. Finally, to integrate species-metabolite association studies utilizing fecal metabolome data with COBRA modeling, we introduce a theoretical framework of how *in silico* calculations refer to *in vivo* statistical associations, resulting in an analysis paradigm that allows for causal inference on metabolome-microbiome relations.

Causal and confounding paths in species-metabolite associations in vivo

Statistical correlation between species abundances and metabolite concentrations in observational data can be either a result of confounding or causation. We discuss first the causal paths by utilizing directed acyclic graphs[12] leading to species-metabolite association by physiological, biochemical, or ecological mechanisms (Figure 1). First, a species can produce or consume a metabolite, directly influencing the metabolite's concentration (direct metabolic causation). Second, a species may produce an intermediate, which then is converted by another microbe into the metabolite under consideration (indirect metabolic causation). This effect can be seen, for example, in microbial bile acid metabolism.[17] Third, a microbe can influence the abundance of another microbe via competition or cooperation,[20] which in return is causally linked to the metabolite (ecological causation). Fourth, a microbe may modulate a physiological factor of the host (for example, inflammation[21]), which then may influence the concentration of the metabolite under consideration (physiological causation). Fifth, a microbe may modulate a behavioral factor, for example, dietary habits,[22] which may impact the metabolite's concentration (behavioral causation) (Figure 1).

However, confounding plays an equally important role as a source of correlation (Figure 1). Physiological factors of the host, such as constipation,[23] may impact the abundance of a microbial species, while also affecting a metabolite (physiological confounding). Thus, these factors may induce correlation between the species abundance and the metabolite, which are otherwise unrelated. Second, behavioral traits of the host, such as diet,[24] may influence microbial abundances and metabolite concentrations (behavioral confounding). In addition, another species may be causally related to the species of interest while impacting the levels of a metabolite (direct ecological confounding). Alternatively, a third factor (microbial, physiological, or behavioral) may induce correlation between the species under consideration and another species, which is causally linked to the metabolite's concentration (indirect ecological confounding). In all these scenarios of confounding, we would observe a correlation between species abundance and metabolite concentrations without any underlying causal relation.

When determining associations by integrating metabolomic and metagenomic data, the various causal and confounding paths are added up to one single association statistic (e.g., the regression coefficient), making statistically significant associations difficult to interpret. Therefore, we need to integrate further information into the interpretation of species-metabolite association to allow for causal inference. Next, we show that COBRA

CellPress
OPEN ACCESS

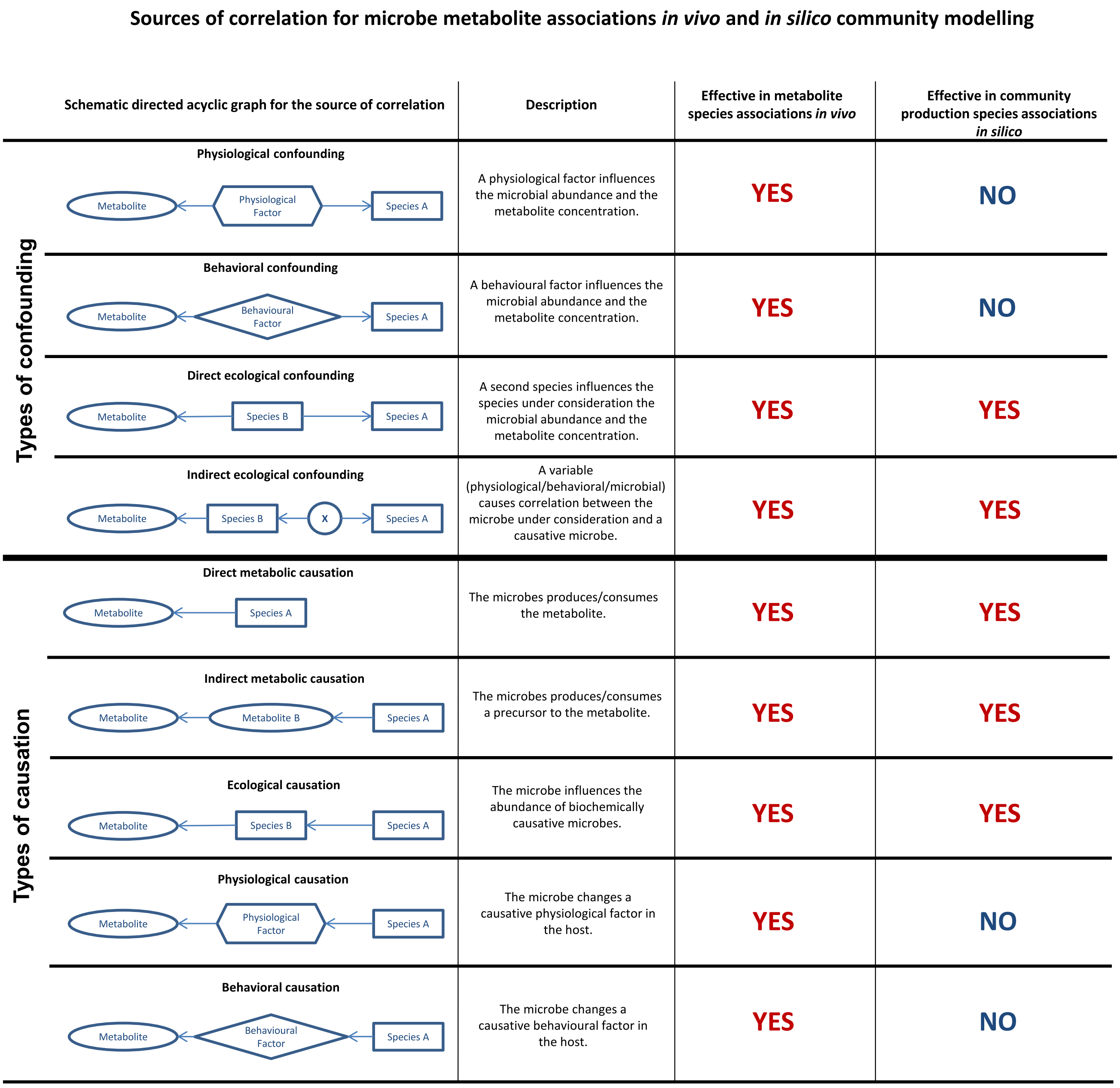

Figure 1. Causal and confounding paths in *in vivo* and *in silico* species metabolite association studies

community models can provide the necessary additional context on the nature of metabolite-species associations to allow for a refined interpretation of association statistics.

Causal and confounding paths in species-metabolite associations in silico

An individual COBRA microbial community model provides the metabolic secretion profile of the microbial community under consideration by calculating maximal net production fluxes from a set of diet constraints, the underlying genome-scale reconstructions of the individual microbes, and the measured microbial community composition from either 16S rRNA or shotgun metagenomic data.[16,25] If we have a population of microbial communities and their corresponding COBRA community models, we can derive an *in silico* flux-species correlation pattern by correlating the species abundances with the overall community net metabolite production capacities. These association patterns, expressed in fluxes rather than in concentrations, can be seen as theoretical counterparts to the *in vivo* species metabolite association patterns from metabolome-microbiome association studies. Importantly, as multiple studies showed, variance in microbial abundance and thus variance in metabolic secretion and consumption capacities influence the metabolic

profiles of the host.[7–9] Thus, if COBRA microbial community models are valid descriptions of the actual microbial activity, then variation in the *in silico* net secretion pattern should translate into *in vivo* variation in metabolite concentrations in the host. However, this assumption needs further theoretical considerations, as COBRA microbial community models do not reflect all causal and confounding paths effective in *in vivo* species-metabolite associations (Figure 1).

COBRA community modeling allows to calculate the direct contribution of each species to each metabolic net production profile of a community, thereby quantifying the direct and indirect metabolic causal effects.[17] In addition, it also allows for quantifying the ecological effects, although no inference on the nature (i.e., causal vs. confounding) of the ecological effects can be made from community modeling alone.[26] In essence, all confounding and causal pathways, which lead to correlation among species abundances, impact the output of COBRA microbial community models, explicitly including ecological causality and the two types of ecological confounding (Figure 1). Crucially, if the diet is held constant across the interrogated population of the computational microbial community models, as done in Baldini et al.[18] and Hertel et al.,[19] all sources for species-metabolite correlation *in silico* lie within the composition of the microbiome, while for *in vivo* species-metabolite associations causal and confounding paths linked to physiological and behavioral variation are also shaping the associating pattern.

Integrating in silico *modeling with metabolome-microbiome association statistics*

Based on these arguments, we can conclude that, in the case of an *in silico* species-metabolite association, the microbiome, at least in the computational model, is causally related to the metabolite under consideration, while no conclusion about causality can be drawn for an *in vivo* association. However, as *in silico* species-metabolite associations are statistical model-based predictions, *in silico* associations alone without empirical evidence remain hypothetical. Hence, combining metabolome-microbiome association studies with COBRA microbial community modeling holds the promise of overcoming limitations associated with the individual paradigms. In essence, for a given metabolite, if the species-metabolite associations *in silico* and *in vivo* systematically correlate, we can conclude that the microbiome is causally related to the metabolite and that the microbial community model is indicative of the net secretion or consumption of the metabolite through the microbiome. If no systematic correlation between *in silico* and *in vivo* association statistics is found, no conclusion can be drawn, as there are many reasons for missing correlation, including a lack of statistical power, measurement error, physiological and behavioral confounding, or incomplete metabolic reconstructions of the microbes. For the same reasons, we should not expect perfect correlation between the two classes of associations, as they do not share all causal and confounding paths.

Theoretical justification via structural equation modeling

For the formalization of the arguments above, we define:

(1) Microbiome community composition $\boldsymbol{a} := (a_1, a_2, \ldots, a_L) \in [0,1]^L$ with $\sum_{l=1}^{L} a_l = 1$
(2) Host characterization $\boldsymbol{h} := (h_1, h_2, \ldots, h_K) \in \mathbb{R}^K$
(3) Community net secretion $\boldsymbol{f} := (f_1, f_2, \ldots, f_J) \in \mathbb{R}^J$ of J metabolite flux
(4) Host metabolome $\boldsymbol{c} := (c_1, c_2, \ldots, c_J) \in \mathbb{R}^J$ of J metabolite concentrations

We will call the vector $(\boldsymbol{a}, \boldsymbol{h}, \boldsymbol{f}, \boldsymbol{c})$ metabolic microbiome-host system. Now, consider that we have sampled N independent and identically distributed (IID) realizations of the vector $(\boldsymbol{a}, \boldsymbol{h}, \boldsymbol{c})_1, (\boldsymbol{a}, \boldsymbol{h}, \boldsymbol{c})_2, \ldots, (\boldsymbol{a}, \boldsymbol{h}, \boldsymbol{c})_N$, describing the case of a cohort with microbiome measurements and host metabolome measurements. Thus, we understand $(\boldsymbol{a}, \boldsymbol{h}, \boldsymbol{c})$ as vectors of random variables with well-defined but unknown distributional characteristics. Utilizing COBRA microbial community modeling with fixed diet constraints, $\boldsymbol{f}$ is approximated by a deterministic known function based on microbiome composition $\boldsymbol{a}$ and the genome-scale reconstructions of the microbes detected in $\boldsymbol{a}$ (see STAR Methods, simulations). In this case, all inter-model variation in $\boldsymbol{f}$ is caused by variation in $\boldsymbol{a}$. For justifying our methodology, we assume that the interrelations can be reasonably approximated by the following structural linear equation system (Figure 2):

$$a_l = u_l + \sum_{k=1}^{K} b_{ah_{lk}} h_k, \text{ leading to } \boldsymbol{a} = \mathbf{B}_{ah}\boldsymbol{h} + \boldsymbol{u} \text{ with } \mathbf{B}_{ah} \in MAT(L,K) \text{ and } \boldsymbol{u} = (u_1, u_2, \ldots, u_L), \quad \text{(Equation 1)}$$

$$f_j = \sum_{l=1}^{L} b_{fa_{jl}} a_l, \text{ leading to } \boldsymbol{f} = \mathbf{B}_{fa}\boldsymbol{a} \text{ with } \mathbf{B}_{fa} \in MAT(J,L), \quad \text{(Equation 2)}$$

$$c_j = \sum_{i=1}^{J} b_{cf_{ji}} f_i + \sum_{k=1}^{K} b_{ch_{jk}} h_k + \varepsilon_j, \text{ leading to } \boldsymbol{c} = \mathbf{B}_{cf}\boldsymbol{f} + \mathbf{B}_{ch}\boldsymbol{h} + \boldsymbol{\varepsilon} \text{ with } \mathbf{B}_{cf} \in MAT(J,J), \mathbf{B}_{ch} \in MAT(J,K), \boldsymbol{\varepsilon} = (\varepsilon_1, \varepsilon_2, \ldots, \varepsilon_J). \quad \text{(Equation 3)}$$

We furthermore impose the following assumptions on the linear structural equations.

(1) $\boldsymbol{u} \perp\!\!\!\perp \boldsymbol{h}$, (stochastic independence between $\boldsymbol{u}$ and $\boldsymbol{h}$),
(2) $\boldsymbol{\varepsilon} \perp\!\!\!\perp \boldsymbol{h}, \boldsymbol{f}$ (stochastic independence between $\boldsymbol{\varepsilon}$ and $\boldsymbol{h}, \boldsymbol{f}$),
(3) $\mathbf{B}_{fa}\mathbf{B}_{ah} = \mathbf{0}$ (orthogonality of $\mathbf{B}_{ah}$ *and* $\mathbf{B}_{fa}$).

Thus, the microbial abundance a_l can be understood as a function of the host and a host-independent factor u_l that is intrinsic to the microbiome. The microbial community net secretion f_j is understood as a function of the microbiome composition and can be modeled as the sum of the individual microbe's metabolite secretions. The coefficients $b_{fa_{lj}}$ are functions of the underlying genome-scale reconstructions and therefore not related to host physiology. The host concentration then is described as a function of the microbial community secretion and the host factors plus an error term ε_j.

While assumptions (1) and (2) are common assumptions about the independence of error terms within structural equations, the third assumption deserves further explanations. The statement

CellPress
OPEN ACCESS

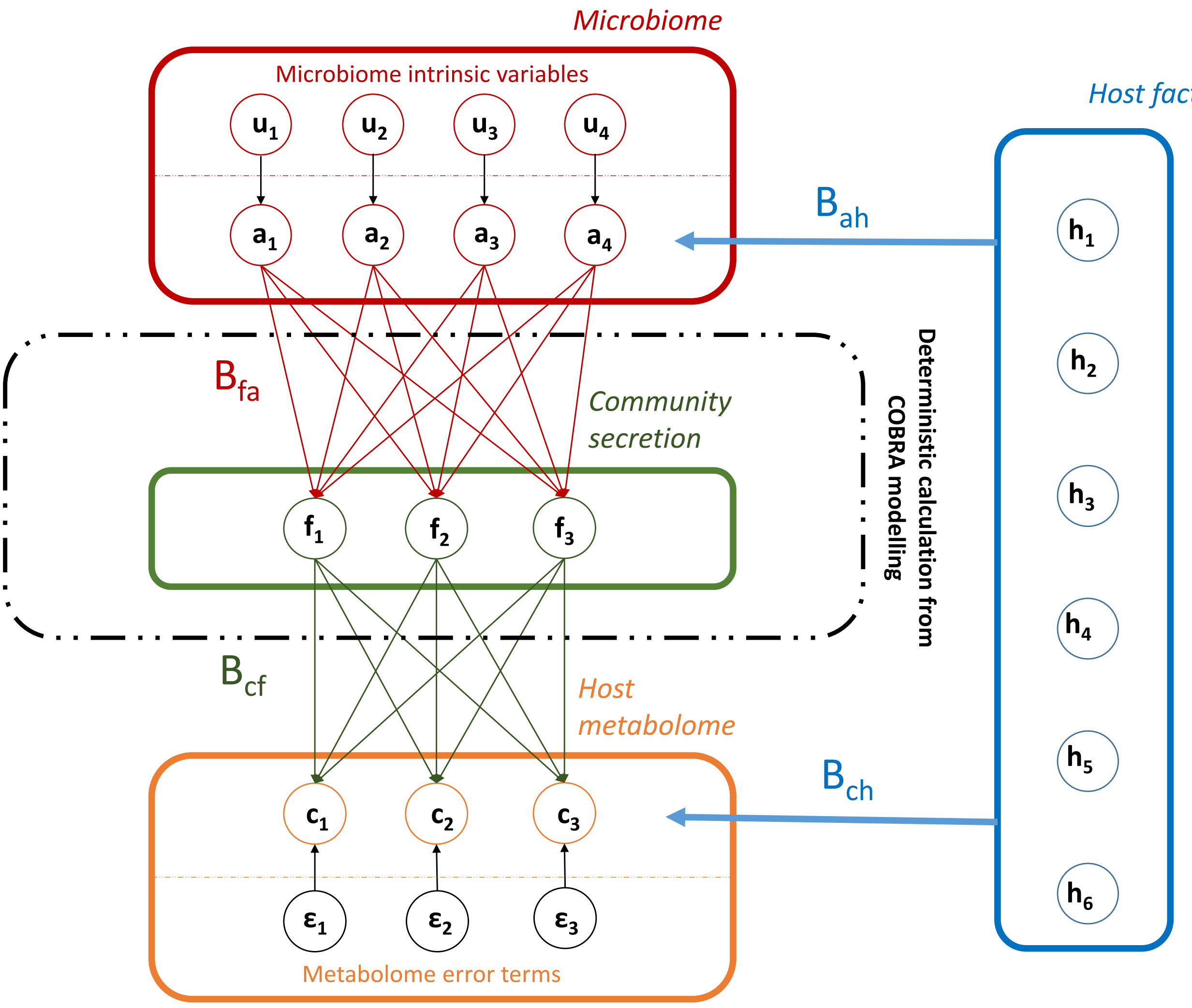

Figure 2. Graphical representation of the structural equation system in an exemplary way describing the interrelations in metabolic host-microbiome systems as conceptualized in this work in the form of a directed acyclic graph

$(a_1, a_2, \ldots, a_4)$, microbiome abundances; $(h_1, h_2, \ldots, h_6)$, host characterization; (f_1, f_2, f_3), community secretion; (c_1, c_2, c_3), host metabolite concentrations.

imposes that the effects of the host factors on the microbiome abundances $b_{ah_{lk}}$ for each column k are orthogonal to the effects of the abundances on the secretion fluxes $b_{fa_{jl}}$ for each row j. Note that the strict orthogonality can be relaxed to approximate orthogonality without difficulties. Assumption (3) is plausible, since otherwise we would state that the modeling parameters derived from COBRA modeling are functions of parameters related to host factors that are not represented in the underlying genome-scale reconstructions of the microbes. In essence, $\boldsymbol{f}$ is stochastically independent of $\boldsymbol{h}$ conditional on $\boldsymbol{a}$, and $\mathbf{B}_{fa}$ carries no information on $\boldsymbol{h}$.

Importantly, from (3) it follows that $\sum_{l=1}^{L} b_{ahlk} = 0$ for all $k \in \{1,2, \ldots,K\}$, since $\sum_{l=1}^{L} a_l = 1$ and $\boldsymbol{u} \perp\!\!\!\perp \boldsymbol{h}$, and thus $COV(\boldsymbol{b_{fa_{j.}}}, \boldsymbol{b_{ah_{.k}}}) = 0$ for all j,k with $\boldsymbol{b_{fa_{j.}}} = (b_{fa_{j1}}, b_{fa_{j2}}, \ldots, b_{fa_{jL}})$ and $\boldsymbol{b_{ah_{.k}}} = (b_{ah_{1k}}, b_{ah_{2k}}, \ldots, b_{ah_{Lk}})$ (see Note S1 for details). The latter result, making use of the compositional nature of microbiome abundance data, explicates that the effects of the abundances on the fluxes should not be systematically correlated to the effects of the host on the abundances.

In the structural Equation 3, a metabolite concentration c_j will be causally related to $\boldsymbol{a}$ if $b_{cf_{ji}} \neq 0$ for at least one secretion flux f_i. Here, we discuss the case $i = j$ and thus the case if the secretion of the same metabolite is causal for the host concentration. In this case, the coefficient $b_{cf_{jj}}$ is the parameter of interest and the matrix $\mathbf{B}_{cf}$ is diagonal. We define,

$$\widehat{b}_{fa_{jl}} = \frac{COV(f_j, a_l)}{VAR(a_l)}, \ \widehat{\boldsymbol{b}}_{\boldsymbol{fa}_{j.}} = \left(\widehat{b}_{fa_{j1}}, \widehat{b}_{fa_{j2}}, \ldots, \widehat{b}_{fa_{jL}}\right) \quad \text{(Equation 4)}$$

(*in silico* association statistics),

which is the vector of regression coefficients of the regression prediction score:

$$\widehat{f}_j = \widehat{b}_{fa_{lj}} a_l + b_{fa0}. \quad \text{(Equation 5)}$$

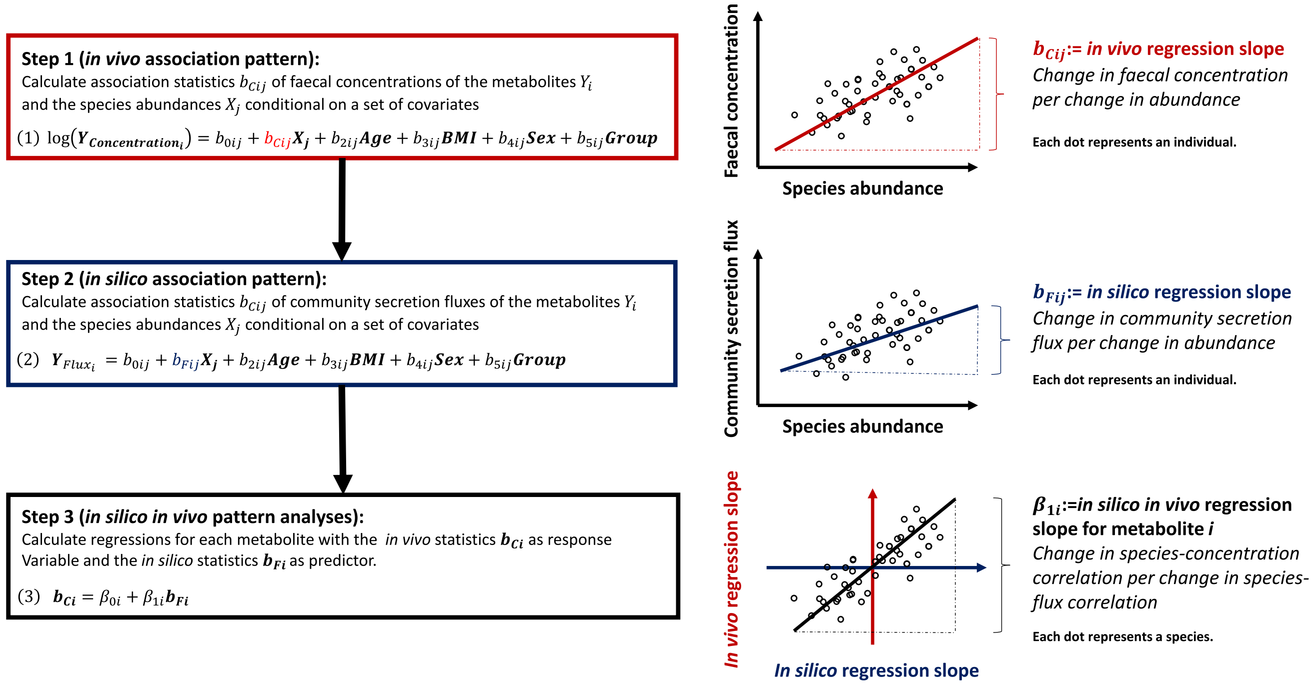

Figure 3. The three steps of *in silico in vivo* association pattern analyses operationalized in terms of linear regression modeling

Second, we define,

$$\widehat{b}_{ca_{jl}} = \frac{\mathrm{COV}(c_j, a_l)}{\mathrm{VAR}(a_l)}, \ \widehat{\boldsymbol{b}}_{\boldsymbol{ca_{j.}}} = \left(\widehat{b}_{ca_{j1}}, \widehat{b}_{ca_{j2}}, \ldots, \widehat{b}_{ca_{jL}}\right)$$
$$\times \text{ (\textit{in vivo} association statistics)}, \quad \text{(Equation 6)}$$

which is the vector of the regression coefficients of the regressions prediction score:

$$\widehat{c}_j = \widehat{b}_{ca_{jl}} a_l + b_{ca0}. \quad \text{(Equation 7)}$$

The central statement now is that for standardized a_l such that $\mathrm{VAR}(a_l) = 1$:

$$\text{If and only if } b_{cf_{jj}} \neq 0, \text{ then } \mathrm{COV}\left(\widehat{\boldsymbol{b}}_{\boldsymbol{ca_{j.}}}, \widehat{\boldsymbol{b}}_{\boldsymbol{fa_{j.}}}\right) \neq 0. \quad \text{(Equation 8)}$$

Thus, testing on $\mathrm{COV}(\widehat{\boldsymbol{b}}_{\boldsymbol{ca_{j.}}}, \widehat{\boldsymbol{b}}_{\boldsymbol{fa_{j.}}}) = 0$ (covariance between *in silico* and *in vivo* association statistics for a given metabolite across the microbiome) the delivers a plausible test, on whether a causal relation between microbiome secretion and host metabolite concentration exist.

To justify this criterion, we can show (see Note S1 for details) that,

$$\mathrm{VAR}(a_l)\widehat{b}_{fa_{jl}} = \mathrm{COV}\left(\sum_{i=1}^{L} b_{fa_{ij}} u_i, u_l\right), \quad \text{(Equation 9)}$$

and

$$\mathrm{VAR}(a_l)\widehat{b}_{ca_{jl}} = b_{cf_{jj}}\widehat{b}_{fa_{lj}} + \mathrm{COV}\left(\sum_{k=1}^{K} b_{ch_{jk}} h_k, \sum_{t=1}^{T} b_{ah_{lt}} h_t\right). \quad \text{(Equation 10)}$$

We see that $\widehat{b}_{ca_{jl}}$ is directly a function of $\widehat{b}_{fa_{lj}}$ if $b_{cf_{jj}} \neq 0$. Thus, if $b_{cf_{jj}} \neq 0$, then $\mathrm{COV}(\widehat{\boldsymbol{b}}_{\boldsymbol{ca_{j.}}}, \widehat{\boldsymbol{b}}_{\boldsymbol{fa_{j.}}}) \neq 0$, except for scenarios with measure zero, where causal pathways exactly cancel each other out (see Spirtes et al.[27] for details).

Now, if $b_{cf_{jj}} = 0$, then,

$$\mathrm{VAR}(a_l)\widehat{b}_{ca_{jl}} = \mathrm{COV}\left(\sum_{k=1}^{K} b_{ch_{jk}} h_k, \sum_{t=1}^{K} b_{ah_{lt}} h_t\right). \quad \text{(Equation 11)}$$

Hence, after standardizing a_l, such that $\mathrm{VAR}(a_l) = 1$, $\widehat{b}_{ca_{jl}}$ is a function of $\boldsymbol{h}$, $\mathbf{B}_{\mathrm{ah}}$, and $\mathbf{B}_{\mathrm{ch}}$. In this case, only confounding pathways through $\boldsymbol{h}$ are effective. As $\boldsymbol{u} \perp\!\!\!\perp \boldsymbol{h}$ and $\boldsymbol{f} \perp\!\!\!\perp \boldsymbol{h} | \boldsymbol{a}$, it follows in this case that $\mathrm{COV}(\widehat{\boldsymbol{b}}_{\boldsymbol{ca_{j.}}}, \widehat{\boldsymbol{b}}_{\boldsymbol{fa_{j.}}}) = 0$.

Note that the standardization of a_l is not a step to make the derivations more convenient, but a necessary step, since both $\widehat{\boldsymbol{b}}_{\boldsymbol{ca_{j.}}}$ and $\widehat{\boldsymbol{b}}_{\boldsymbol{fa_{j.}}}$ will otherwise depend on the vector of abundance variances $\mathrm{VAR}(a_l)$, and thus could bias $\mathrm{COV}(\widehat{\boldsymbol{b}}_{\boldsymbol{ca_{j.}}}, \widehat{\boldsymbol{b}}_{\boldsymbol{fa_{j.}}})$. Importantly, the *in silico* association statistics $\widehat{\boldsymbol{b}}_{\boldsymbol{fa_{j.}}}$ are containing information on the community structure, as the covariance pattern of microbiome abundances is represented in the form of $\mathrm{COV}(\sum_{i=1}^{L} b_{fa_{ij}} u_i, u_l)$. The latter result means that *in silico* association statistics are functions of the covariance across the randomly sampled microbiome compositions $\boldsymbol{a}$.

In silico in vivo association pattern analyses

These considerations lead to a methodological paradigm, which we call "*in silico in vivo* association pattern analyses," which integrates population statistics with COBRA modeling. This paradigm requires metagenomic data quantifying the microbiome at a body site, corresponding metabolomic measurements from the host, a collection of genome-scale metabolic reconstructions, such as AGORA[4] or AGORA2,[28] for the generation of the COBRA microbial community models, and adequate metadata for controlling for important covariates, e.g., age, sex, and body mass index. Conceptually, *in silico in vivo* association pattern analyses can be defined by three steps (Figure 3).

(1) Step 1 (*in vivo* association pattern):

Calculate association statistics for the associations of metabolite concentrations with the standardized species abundances conditional on a set of covariates.

(2) Step 2 (*in silico* association pattern):

Calculate association statistics for the associations of microbial community net metabolite secretion fluxes as determined by COBRA community modeling and the measured standardized species abundances conditional on a set of covariates.

(3) Step 3 (*in silico in vivo* pattern analyses):

Calculate regressions for each metabolite with the significant *in vivo* metabolite-species association statistics as response variable and the corresponding *in silico* association statistics as predictor. Test the resulting regression coefficients on zero. A significant regression coefficient, and thus a significant *in silico in vivo* association pattern, indicates that the microbiome is causally related to the metabolite under consideration.

The concrete statistical operationalization (i.e., which covariates to include, questions of statistical model parametrizations, and so on) is dependent on the study design. The most canonical way to retrieve association statistics is via a set of linear regression models (Figure 3). However, other statistical paradigms could be used as well. For example, beyond correlating *in vivo* and *in silico* association statistics in step 3, one may compare the sign of the two types of association statistics via hypergeometrical tests. Given the unknown distributional properties of *in vivo in silico* association pattern, and in particular the stochastic dependencies between association statistics stemming from multivariate compositional data, we propose to utilize permutation procedures for significance testing.

Importantly, the *in silico* association statistics contain information on the microbial community structure as the covariance pattern of microbiome abundances is represented in them.

Summary of the theoretical part

In summary, we have established that *in vivo* species-concentration association statistics and *in silico* species-flux association statistics theoretically share certain sources of variance, while they do not completely overlap. Our theoretical considerations led consequentially to the conclusion that for metabolites, whose concentrations in the host are systemically influenced by variance of the gut microbiome composition, we should see a substantial correlation between *in vivo* species concentration association statistics and *in silico* species flux association statistics. These considerations were then formalized in terms of linear structural equations.

This hypothesis of correlating association statistics is testable via integrating metabolome data with microbial community models based on metagenomic quantifications of the microbiome, as outlined in the section on *in silico in vivo* association pattern analysis. The most direct test can be performed by integrating computational community models of the gut microbiome with fecal metabolome data, being physiologically closest to the gut microbiome, but the outlined principles also hold for other compartments and body sites (e.g., the oral microbiome and the saliva metabolome). Importantly, the hypothesis requires COBRA microbial community models to be fundamentally valid in their capability to predict actual metabolic activity. Testing the correlation of *in vivo* and *in silico* species-metabolite association statistics delivers a fundamental model test of COBRA community modeling regarding its ability to reflect the real metabolic activity of the gut microbiome.

Empirical results

For testing the theoretical framework outlined above, we utilized fecal metabolome and metagenomic data from Yachida et al.[7] First, we mapped the measured fecal metabolome data, reporting the absolute concentrations for 450 metabolites, onto the AGORA collection of 818 microbial genome-scale reconstructions.[4] We found that 106 metabolites were measured by the metabolome data and had a corresponding metabolite exchange reaction in at least one microbial reconstruction. From these 106 metabolites, 54 had values above the limit of detection in the fecal metabolome for at least 50% of all samples and, thus, they were included in the subsequent analyses (Figures 4A and 4B). Second, we mapped the relative metagenomic quantifications that had been reported by Yachida et al.[7] based on the MetaPhlAn2 pipeline[28] onto the AGORA collection.[4] Of 623 measured taxa, 517 were named microbial species. Of those, 362 (97.51% ± 3.32% of the total abundance) were included in AGORA (Figure 4C). Next, applying personalized COBRA microbial community modeling,[16,25] we derived for each of the 54 metabolites the net production capacity under an *in silico* average Japanese diet for 347 individuals who had metabolome measurements (n = 220 colorectal cancer cases; n = 127 healthy controls). The analysis sample consisted of n = 347 individuals (Figure 4D). We then calculated the *in vivo* species-concentration associations using the fecal metabolite measurements via multivariable regressions, deriving the full fecal species-concentration association pattern (step 1). Using the *in silico* metabolic profile, we correlated the abundance of each species with the overall net community production capacity analogously (step 2), giving rise to an *in silico* species-flux association pattern (step 3). The species-association patterns were generated for all 148 microbial species (Figure 4C), which were found in at least 10% of all samples, resulting overall in 7,992 species-metabolite associations. We generated two types of *in silico in vivo* association pattern, (1) one pattern with respect to the species presence and (2) one pattern with respect to the

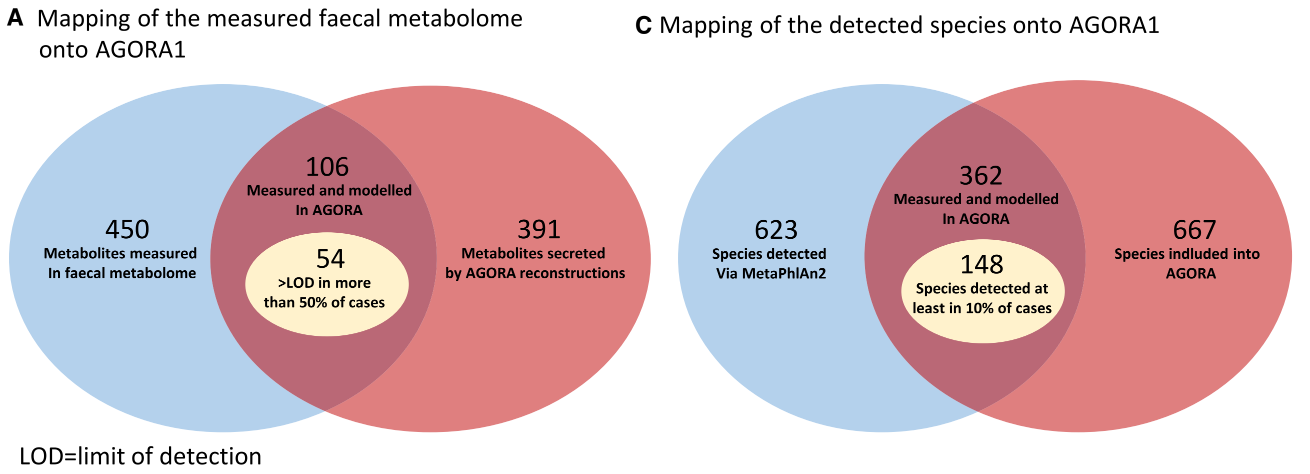

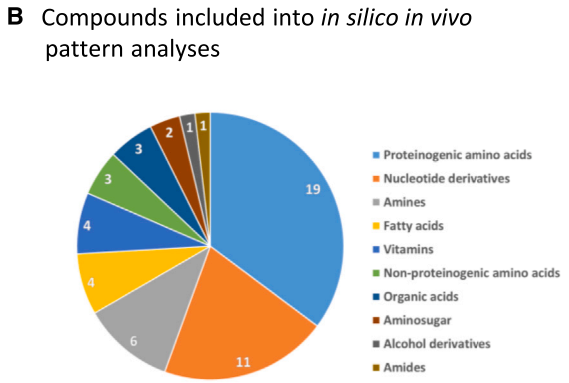

D Sample characteristics of the study

	CRC Patients (n=220)	Healthy controls (n=127)	p-value
**Age, mean (SD)**	62.42(10.05)	64.65(10.55)	0.055[a]
**BMI, mean (SD)**	23.05 (3.42)	22.96(2.78)	0.802[a]
**Female, %**	39.55%	44.09%	0.429[b]
**Species richness, mean (SD)**	65.12(14.80)	61.76 (14.38)	0.036[a]
**# Metabolites produced, mean (SD)**	210.06 (7.16)	209.74(7.55)	0.700[a]
**# Reactions in community models, mean (SD)**	83980.91 (19762.44)	79482.24 (18283)	0.033[a]

CRC=Colorectal cancer, SD=Standard deviation, [a]p-value from Welch's t-tests, [b]p-value from Fisher's exact test

Figure 4. Overview on the utilized empirical dataset
(A) Mapping of the measured fecal metabolome onto the AGORA resource of gut microbial metabolic reconstructions.[4]
(B) Compounds included in the *in silico in vivo* pattern analyses.
(C) Mapping of the detected microbial species onto AGORA.
(D) Sample characteristics of the utilized study dataset.

species abundance. For both patterns, we analyzed the agreement in the sign and the value of the *in silico* and *in vivo* association statistics, resulting overall in four sets of *in silico in vivo* comparisons.

In vivo *species presence metabolite association patterns are predicted by COBRA community models*

In total, we found 1,737 associations between fecal metabolite concentrations and species presence with $p < 0.05$, and 1,210 associations with a false discovery rate (FDR) < 0.05, with putrescine and tryptophan having the highest number of significant species presence associations (Table S1). For adenosine, spermidine, riboflavin, and dodecanoic acid, we found less than 10 species presence associations with $p < 0.05$ (Table S1). *In silico* species presence association pattern significantly predicted the sign of *in vivo* species-metabolite associations for 16 metabolites according to permutation tests (Figure 5; Table S3). Regarding the value of the *in vivo* association statistics, COBRA-based *in silico* association statistics correlated significantly for 19 metabolites according to permutation tests ($p < 0.05$) with their corresponding *in vivo* association statistics (Figure 5; Table S3). Noteworthy, sign prediction has low statistical power if there is little variance in the signs of the *in vivo* associations (e.g., all or nearly all associations are positive or negative).

Importantly, for four metabolites (spermidine, guanine, N-acetyl-D-glucosamine, and glutarate) *in silico* associations were systematically inversed to the *in vivo* associations (Figure 6; Table S4). In all these cases, microbial exchange directions were noted to be bidirectional in the AGORA resource. The easiest explanation for this pattern is therefore to interpret the secretion potential as a metric of net consumption. In an earlier work,[26] the validity of this interpretation was demonstrated for glutarate.

In vivo *species abundance metabolite association patterns are predicted by COBRA microbial community models*

Calculating the *in vivo* abundance fecal concentration association pattern via regressions, we found 1,310 abundance concentration associations with an FDR < 0.05 and 1,800 associations

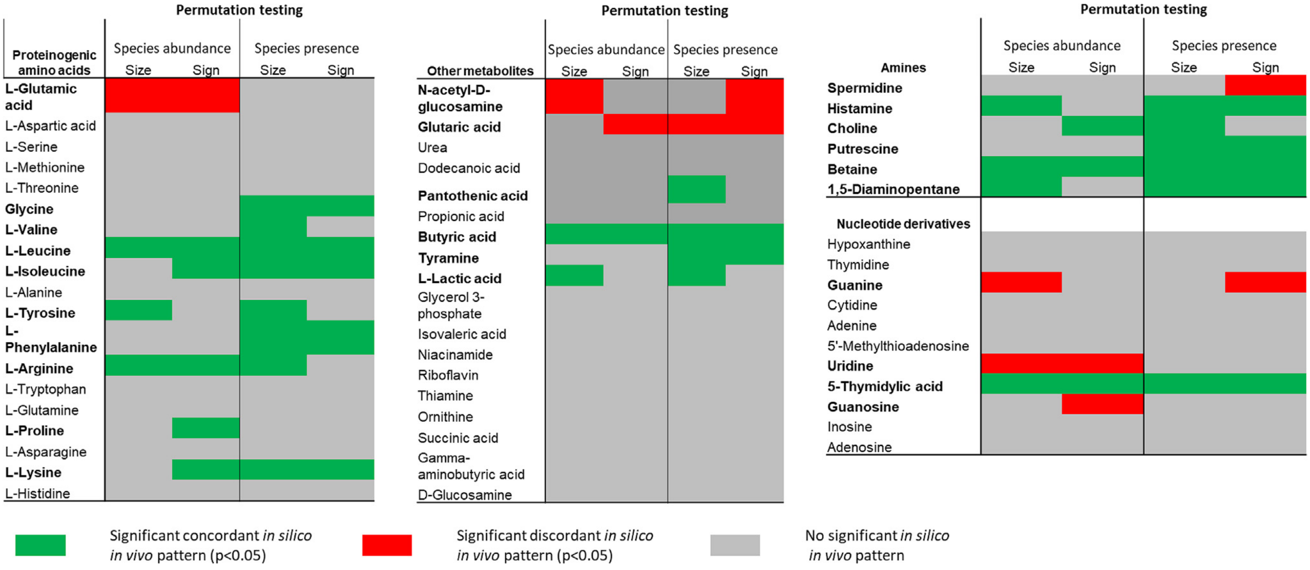

Figure 5. Overview on the *in vivo in silico* pattern association analyses for 54 fecal metabolites, results shown for both parametric hypothesis testing and permutation testing

(A) Discordance (significant, inverse association between *in vivo* and *in silico* association statistics) and concordance (significant, positive association between *in vivo* and *in silico* association statistics) for the 54 metabolites separated by metabolite class.

with $p < 0.05$ with putrescine, alanine, and tryptophan showing the largest numbers of species associations (Table S2). With respect to the sign of association, the *in silico* association pattern significantly predicted the sign for 13 metabolites using permutation tests (Figure 5; Table S4). With respect to the value of the association statistics, 13 metabolites showed a significant correlation between *in vivo* and *in silico* association statistics with an FDR < 0.05 (Figure 5; Table S4). As with the species presence association pattern analyses, we observed systematically inversed associations for a range of metabolites, indicating that maximal net secretion fluxes represent net consumption in these cases.

In silico in vivo *association pattern analyses reveals broad causal microbiome-metabolome relations*

Overall, *in silico* and *in vivo* association statistics significantly related to each other for 26 out of the 54 metabolites in at least one domain (i.e., value or sign for species abundance or species presence association patterns, respectively) in permutation tests. Importantly, from 23 significant association patterns, 7 were consistently discordant (negative correlations between *in vivo* and *in silico* association statistics). The various degrees of correlations across the metabolites between *in silico* and *in vivo* association statistics can range from significant (isoleucine, N-acetyl-D-glucosamine, butyrate) and insignificant (propionate), concordant (isoleucine, butyrate) and discordant (N-acetyl-D-glucosamine) association patterns (Figure 6). All significant patterns per metabolite were consistent, as the computed fluxes cannot indicate net consumption (discordant pattern) and net secretion (concordant pattern) at the same time. Potential inconsistencies in concordant and discordant pattern within one metabolite could hint at false positive results or model misspecifications.

In conclusion, as we found clear pattern of correlation between *in silico* and *in vivo* association statistics, *in silico in vivo* association pattern analyses revealed substantial causal contributions through the gut microbiome for fecal concentrations of 26 metabolites from a wide range of classes (Figure 5). However, *in silico in vivo* association patterns were more pronounced for amino acids and amines than for nucleotides, hinting at a higher variance contribution of the microbiome to the fecal metabolome in the domain of amino acid metabolism than in nucleotide metabolism.

In conclusion, *in silico in vivo* association pattern analysis allows for a characterization of the component in the host metabolome that is causally related to the microbiome.

DISCUSSION

The microbiome contributes essentially to the human metabolism by providing essential nutrients, such as vitamins and short-chain fatty acids, which would be inaccessible otherwise.[29] Numerous studies have been conducted to shine a light on the complex metabolic host-microbiome interplay, generating insights from a diverse range of paradigms spanning experimental work,[30–32] computational and theoretical studies,[11,33] and statistical interrogation into the dependence patterns of observational multi-omics datasets.[13,34] Herein, we bridge the gap between population statistics and *in silico* COBRA microbial community modeling to facilitate causal inference in metabolome-microbiome observational data.

For identifying causal relationships in observational data, either full information on relevant covariates[35] or plausible instrumental variables (e.g., as utilized in Mendelian randomization[36]) must be available. In the case of microbiome-metabolome

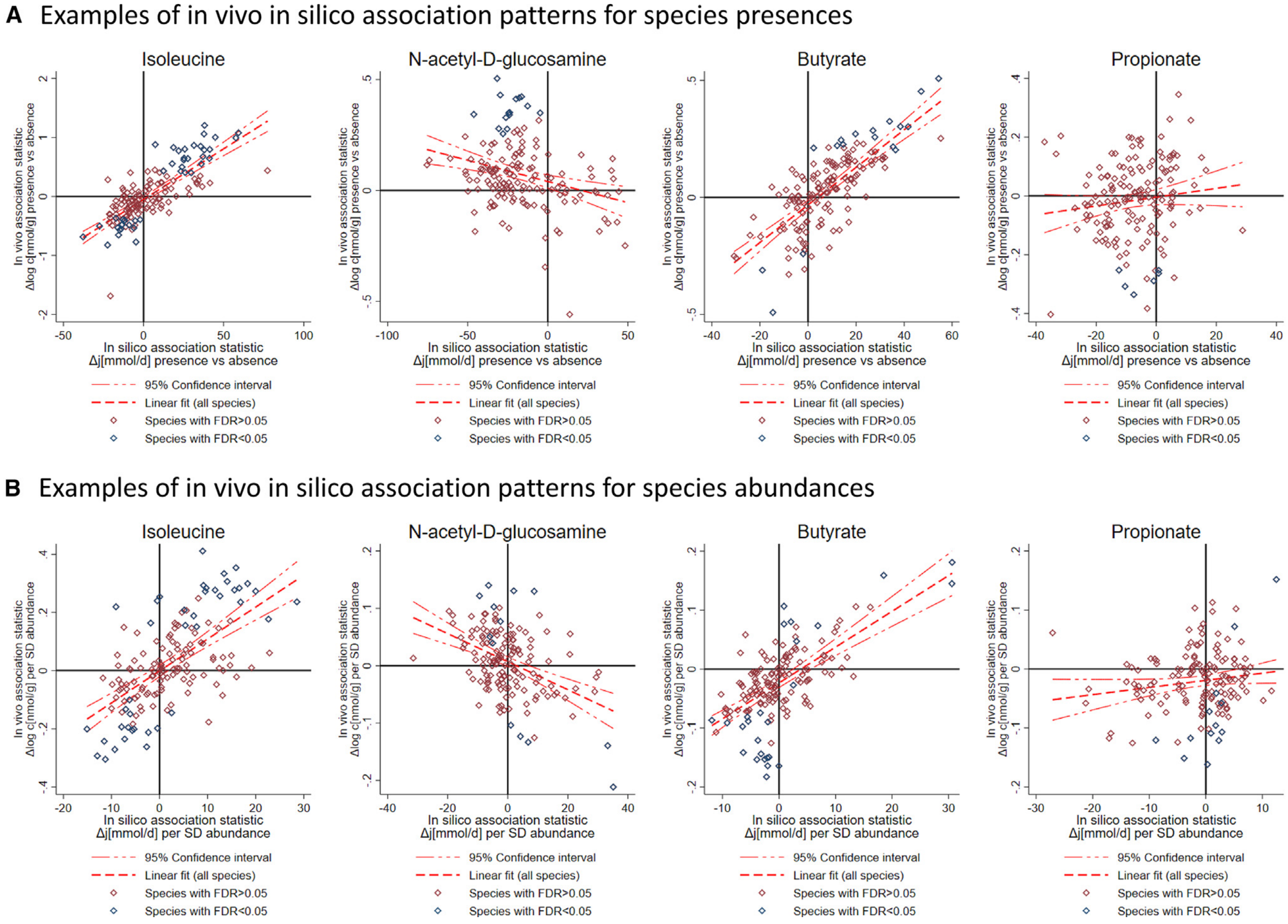

Figure 6. Examples for *in silico in vivo* association patterns with regression line and 95% confidence intervals

Each dot represents a species with the x axis denoting the *in silico* association statistic and the y axis denoting the *in vivo* association statistics. Each plot is based on 296 regressions (two times 148 species). Association patterns for isoleucine (concordant), N-acetyl-D-glucosamine (discordant), and butyrate (concordant) are significant, while for proprionate no significant pattern could be identified. Significance of pattern is determined by a significant slope of the regression line and significant sign (dis)agreement through permutation testing.

association studies, information on relevant covariates, such as diet, is typically missing and often difficult to assess reliably.[13] Moreover, while certain genetic variants have been shown to be associated with the microbiome,[37] the host genetic signature is modest at best[38] making the identification of suitable genetic instrument variables to perform Mendelian randomization difficult. Therefore, metabolome-microbiome associations are challenging to interpret in causal terms and, indeed, simulation studies of microbial communities suggest that naive interpretation would lead to a high level of false positives.[11] However, we demonstrate that we can overcome these difficulties by systematically integrating *in silico* COBRA community modeling into statistical analyses of metabolome-microbiome datasets. On a conceptual level, COBRA community modeling delivers the additional biological context needed to disentangle whether the microbiome is causally related to a metabolite or not.

Systematic *in silico in vivo* association pattern analyses revealed that a wide range of the examined metabolites are causally related to the microbiome (26 out of 54, Figure 5). The causal signature was especially clear for amines and proteinogenic amino acids while, relatively speaking, less pronounced for nucleotides. The weak signature for nucleotides could indicate a higher amount of host contributions to the fecal nucleotide concentrations but may also point toward incomplete representation of microbial nucleotide metabolism in the genome-scale reconstructions. As with all knowledge-based methodologies, well-researched pathways, such as amino acid metabolism, may be better represented than other pathways, leading to clearer signals in the *in silico in vivo* association pattern. Importantly, the predictive power of COBRA microbial community modeling regarding *in vivo* species metabolite associations delivers a strong proof of concept that community models based on genome-scale reconstructions indeed result in quantifications of the actual metabolic activity of microbial communities. It should be noted that the presented results are based on pan-species modeling, which neglects strain-specific differences. It is, therefore, conceivable that strain-resolved modeling may improve the detection of causal relationships. Furthermore,

while working reasonably well for a range of metabolites, the chosen operationalization of flux variability analysis[15,39] may not be universally useful for quantifying metabolite-species relations across all metabolic functions. However, the general methodology of *in silico in vivo* association pattern analysis is not constrained to flux variability analysis. In contrast, it can be performed with any plausible readout of microbiome community models on the level of fluxes.

We analyzed both species presence as well as species abundance. On the surface, this is contra-intuitive, since information on the presence of a species is represented in the abundance data as well. The problem, however, is that statistical regression modeling treats abundances as continuous variables, while in COBRA modeling absence of a microbe leads to changes in the stoichiometric matrix of the model and, therefore, to qualitative changes in aspects of the model (e.g., the null space of the stoichiometric matrix may have different dimensionalities dependent on the presence of a species). When integrating statistical modeling and COBRA modeling, presence-absence analysis is therefore not redundant to abundance analysis but complementary.

We understand "causally related" in terms of the frameworks of Pearl.[12] It should not go unnoticed that the theory of causal statistics, built on the backbone of directed acyclic graphs (Figure 3 is an example), is based on strong assumptions, which can be justifiably challenged,[40] especially in dynamic systems.[41] Moreover, the chosen operationalization of a causal relation can be contra-intuitive. For example, it can be that a microbial community produces a metabolite and that alterations in the community composition changes the production rate without affecting substantially the concentration in the host for various reasons (i.e., saturated transport kinetics). In this case, the microbiome would not be causal for variation in the host, while being causal for the production of the metabolite. This observation represents one of the reasons why *in silico* modeling alone is not sufficient for determining causal relations between metabolite concentrations in the host and species abundances. To give one example, we could not identify a significant *in silico in vivo* association pattern regarding propionate (Figure 6), a short-chain fatty acid known to be produced by the gut microbiome. In the case of strong intra- and interpersonal variation in propionate absorption in the gut (i.e., colonocytes), the influence of the gut microbiome on fecal concentrations may be minor in terms of variance. This result additionally indicates that fecal propionate concentrations may not be good proxies for microbial propionate secretion. In contrast, *in silico in vivo* association pattern analysis was remarkably successful regarding butyrate (Figure 6; Tables S3 and S4), another short-chain fatty acid produced by the microbiome, highlighting that fecal butyrate pools are good indicators of the microbial community butyrate production. The examples of propionate and butyrate show the value of *in vivo in silico* association pattern analyses to determine which fecal concentrations can serve as good biomarkers of microbial metabolic activity. This discussion also highlights the duality of fluxes and concentrations.[42] While concentrations are functions of fluxes, concentrations cannot be equated with fluxes. The outlined methodology, however, gives an empirical criterion, when fluxes from COBRA modeling can be seen as good proxies for host concentrations and when not.

It is worth noting that *in silico in vivo* pattern analyses, strictly speaking, allow only for an inference on whether the microbial community as a whole is causally related to a metabolite. Also, as COBRA microbial community modeling, at least in the herein applied form, cannot differentiate between ecological causation and ecological confounding,[26] causal inference on the species level, strictly speaking, is not possible. One may further argue that causal inference on single species is not sensible due to systems nature of microbial communities, making the concepts of causal inference on the species level more difficult to apply. Nonetheless, *in silico in vivo* association pattern analyses can give insights into individual species-metabolite relations. First, one can calculate the direct contribution of a species to the net secretion potentials.[17] If the species under consideration also shows a significant species-concentration association, one may argue with some justification that a causal relationship was identified. However, one must be careful, as a species can have a positive direct contribution to the net secretion potentials, while displaying large negative ecological effects. An example of this was given in Hertel et al.,[26] where *Fusobacterium* sp. were demonstrated to contribute small amounts of butyrate to fecal butyrate pools, while having large deleterious effects on community butyrate production. In this case, low abundance of prominent butyrate producers, such as *F. prausnitzii*, in *Fusobacterium* sp.-containing communities resulted in an overall negative impact of *Fusobacterium* sp. on community butyrate production.[26]

The latter observation highlights that randomly sampled populations of COBRA microbial community models allow for analysis routes that are impossible to conduct on synthetic microbial communities. For example, synthetic *in silico* microbial communities currently lack abundance covariance structures that could be seen as representative for *in vivo* populations of microbiome-host systems. While such synthetic communities are suitable tools to understand direct and indirect species production effects, ecological effects, in particular in relation to host factors, cannot be correctly represented. Thus, the effects of a microbe on a community secretion flux in synthetic communities is an incomplete proxy for causal species-metabolite relationships.

An important aspect of microbiome modeling via COBRA is the setting of the diet constraints. In the context of this methodology, the diet constraints are held constant across all models, such that the only source of variation in fluxes is variation in community composition. Testing variegations in diet constraints on their effect on *in silico in vivo* pattern is beyond the scope of this work but is possible in principle. Note, however, that this will complicate causal inference, since a second source of variance is introduced with its own confounder structure attached. In previous research,[43] changing the diet constraints to an average European diet did not affect the modeling results in a major way. Nevertheless, the presented empirical results should be understood as an investigation of causal metabolite-microbiome relations under a Japanese diet and may not generalize to all possible diet constraints.

Other potential insights in future studies could be won by analyzing outliers in the *in silico in vivo* association pattern. Outlier species (e.g., species having, for example, very strong *in vivo* associations, while having low *in silico* associations values) may indicate physiological and behavioral influences

Table 1. Classification of reasons for missing *in silico in vivo* association pattern

Statistical	Biological	COBRA modeling based
Low sample size	low/zero net contribution of the microbiome to metabolite pools	incomplete reconstructions
Measurement error	large variance in metabolite levels due to host's physiology	gene mis-annotations
Neglected nonlinearity	large variance in metabolite levels due to diet variation	wrong directionality of transport reactions
Violations against assumptions, such as the IID or normality assumptions	systematic confounding by behavioral or physiological factors	mis-specified constraints
Neglected interaction terms	host-microbiome feedback loops	systematic error due to the steady-state assumptions
–	–	optimization problem solved by flux balance/variability analysis does not reflect community behavior

IID, independent and identically distributed.

on metabolite-species associations not reflected with COBRA microbial community modeling. Thus, they could be targets for further investigations in the direction of the host's physiology and behavior, e.g., using whole-body metabolic models.[44] On the other hand, outliers may also indicate incomplete genome-scale reconstructions. Thus, outlier analyses within *in silico in vivo* association pattern analysis holds promise for increasing the knowledge base and pointing toward species indicative of underlying physiological or behavioral processes.

For 28 metabolites (Figure 5), we could not identify significant *in silico in vivo* association patterns. As already sketched out in the theoretical results part, reasons for missing *in silico in vivo* association pattern can be manifold, making it impossible to disentangle the various possibilities and to conclude that the microbiome has no systematic influence on those metabolites. Table 1 categorizes the different factors leading to missing association pattern into (1) statistical, (2) biological, and (3) model-based factors. Importantly, we are dealing with two types of modeling (statistical modeling and COBRA modeling), each entailing their own set of assumptions. In particular, the need of sufficient statistical power for trustworthy estimation of *in vivo* metabolite-species associations means that *in silico in vivo* association pattern analyses is bound to studies with medium to large sample sizes. Concrete sample size requirements are difficult to give, as they depend on data quality and study design, but the presented analysis on 347 individuals with metabolome and metagenome data was sufficient for comprehensive analyses of *in silico in vivo* association pattern.

In contrast to pure machine learning approaches, our approach includes through genome-scale metabolic reconstructions a wealth of knowledge into the statistical analysis, avoiding "conceptual overfitting."[45] Basically, conceptual overfitting means that machine learning is blind to whether a source of covariation is due to confounding or due to causality. Maximizing statistical model fit can, therefore, lead to parametrizations and statistical models, which may technically show the best fit, but do not approximate what we are interested in on a conceptual level in terms of biology. In the case of metabolome-microbiome studies, we are often interested in the part of the metabolome, which is causally influenced by the microbiome. As our work shows, *in silico in vivo* association pattern analyses is capable of characterizing the part of the fecal metabolome causally related to the microbiome. In contrast, machine learning algorithms will deliver on the question: which part of the metabolome covariates with the microbiome regardless of whether the covariation is caused by confounding or causation?

Regardless of future improvements, the successful characterization of metabolite-microbiome relations in causal terms from association patterns shows promise for applications, where biomarkers of microbial functions are needed. For example, in pre- and probiotic interventions aiming at improving the butyrate production may be supported by butyrate community secretion derived from COBRA modeling. Importantly, those *in silico* biomarkers can be superior to using butyrate quantification in the feces, as butyrate measurements in the host are also influenced by microbiome unrelated factors, lowering arguably the statistical power to detect intervention effects.

Conclusions

We presented a theoretical framework integrating population statistics with COBRA microbial community modeling for causal inference on microbiome-metabolome relations. We then showed the feasibility and validity of our approach on an empirical dataset, consisting of 347 individuals with fecal metabolome and metagenomics data. Conceptually, we validated thereby a methodological framework to incorporate formalized knowledge about microbial biology in the form of genome-scale models into statistical association analyses, bridging two major paradigms of systems biology. The successful identification of significant *in silico in vivo* association patterns for 26 metabolites highlights the validity of COBRA microbial community models as a theoretical model for actual microbial metabolic activity. Importantly, the prediction of *in vivo* species metabolite associations was achieved without training the COBRA community models on the given metabolome dataset. Overall, this study highlights the value of integrating

knowledge-based and data-driven procedures to overcome the limitations of each paradigm alone.

Limitations of the study

Limitations of the introduced methodology lay within the limited possibilities to perform causal inference on the level of individual species, and in the non-representation of behavioral and physiological causality, where the microbiome influences physiological or behavioral attributes of the host and, thereby, the metabolome. The latter aspect may be partially rectified in future studies by introducing personalized diet constraints and comprehensive whole-body modeling[44] for a more holistic picture of host-microbiome metabolic interactions. One particular limitation arises from the use of pan-species modeling, and strain-level modeling may improve the detection of causal metabolite-microbiome relations, where pan-species modeling fails to reflect inter-species metabolic variability. As this method integrates COBRA modeling with statistical association statistics, certain limitations arising from the individual paradigms propagate to *in vivo in silico* association pattern analysis, such as the steady-state assumption underlying COBRA community models. Most noteworthy, statistical power must be sufficient for robust identification of *in vivo* associations, and statistical models need to be correctly specified, and, thus, the methodology is unlikely to be informative in small sample sizes. The modeling relies on the IID assumption and should be treated with care in data situations, where IID cannot be assumed. The formal justification of the paradigm is based on linear structural equation and, thus, it is unclear how the paradigm performs in the presence of nonlinearities.

STAR★METHODS

Detailed methods are provided in the online version of this paper and include the following:

- KEY RESOURCES TABLE
- RESOURCE AVAILABILITY
  - Lead contact
  - Materials availability
  - Data and code availability
- METHOD DETAILS
  - Study sample
  - Construction of sample-specific gut microbiota models
  - Simulations
- QUANTIFICATION AND STATISTICAL ANALYSIS
  - Statistical operationalization of *in vivo* in silico association pattern analyses
  - Species presence *in vivo* association studies
  - Species presence *in silico* association studies
  - Species abundance *in vivo* association studies
  - Species abundance *in silico* association studies
  - *In silico in vivo* association pattern analyses

SUPPLEMENTAL INFORMATION

Supplemental information can be found online at https://doi.org/10.1016/j.crmeth.2023.100615.

ACKNOWLEDGMENTS

This study was funded by grants from the European Research Council (ERC) under the European Union's Horizon 2020 research and innovation programme (grant agreement no. 757922) to I.T., the National Institute on Aging (grant nos. 1RF1AG058942 and 1U19AG063744), and the Science Foundation Ireland (grant no. 12/RC/2273-P2). J.H. received additional funding from the Deutsche Forschungsgesellschaft (DFG) (grant no. HE 9198/1-1).

AUTHOR CONTRIBUTIONS

J.H. and I.T. designed the study. J.H. and I.T. wrote the manuscript. J.H. and D.F. performed the statistical analyses. J.H. developed the theoretical concepts. A.H. constructed the microbial community models and performed the community modeling. All authors reviewed and approved the final manuscript.

DECLARATION OF INTERESTS

The authors declare no competing interests.

INCLUSION AND DIVERSITY

We support inclusive, diverse, and equitable conduct of research.

Received: July 18, 2022
Revised: May 23, 2023
Accepted: September 20, 2023
Published: October 16, 2023

REFERENCES

1. Rowland, I., Gibson, G., Heinken, A., Scott, K., Swann, J., Thiele, I., and Tuohy, K. (2018). Gut microbiota functions: metabolism of nutrients and other food components. Eur. J. Nutr. *57*, 1–24. https://doi.org/10.1007/s00394-017-1445-8.
2. Thaiss, C.A., Zmora, N., Levy, M., and Elinav, E. (2016). The microbiome and innate immunity. Nature *535*, 65–74. https://doi.org/10.1038/nature18847.
3. Weiss, G.A., and Hennet, T. (2017). Mechanisms and consequences of intestinal dysbiosis. Cell. Mol. Life Sci. *74*, 2959–2977. https://doi.org/10.1007/s00018-017-2509-x.
4. Magnúsdóttir, S., Heinken, A., Kutt, L., Ravcheev, D.A., Bauer, E., Noronha, A., Greenhalgh, K., Jäger, C., Baginska, J., Wilmes, P., et al. (2017). Generation of genome-scale metabolic reconstructions for 773 members of the human gut microbiota. Nat. Biotechnol. *35*, 81–89. https://doi.org/10.1038/nbt.3703.
5. Liu, H., Chen, X., Hu, X., Niu, H., Tian, R., Wang, H., Pang, H., Jiang, L., Qiu, B., Chen, X., et al. (2019). Alterations in the gut microbiome and metabolism with coronary artery disease severity. Microbiome *7*, 68. https://doi.org/10.1186/s40168-019-0683-9.
6. Menni, C., Zhu, J., Le Roy, C.I., Mompeo, O., Young, K., Rebholz, C.M., Selvin, E., North, K.E., Mohney, R.P., Bell, J.T., et al. (2020). Serum metabolites reflecting gut microbiome alpha diversity predict type 2 diabetes. Gut Microb. *11*, 1632–1642. https://doi.org/10.1080/19490976.2020.1778261.
7. Yachida, S., Mizutani, S., Shiroma, H., Shiba, S., Nakajima, T., Sakamoto, T., Watanabe, H., Masuda, K., Nishimoto, Y., Kubo, M., et al. (2019). Metagenomic and metabolomic analyses reveal distinct stage-specific phenotypes of the gut microbiota in colorectal cancer. Nat. Med. *25*, 968–976. https://doi.org/10.1038/s41591-019-0458-7.
8. Zierer, J., Jackson, M.A., Kastenmüller, G., Mangino, M., Long, T., Telenti, A., Mohney, R.P., Small, K.S., Bell, J.T., Steves, C.J., et al. (2018). The fecal metabolome as a functional readout of the gut microbiome. Nat. Genet. *50*, 790–795. https://doi.org/10.1038/s41588-018-0135-7.

9. Visconti, A., Le Roy, C.I., Rosa, F., Rossi, N., Martin, T.C., Mohney, R.P., Li, W., de Rinaldis, E., Bell, J.T., Venter, J.C., et al. (2019). Interplay between the human gut microbiome and host metabolism. Nat. Commun. *10*, 4505. https://doi.org/10.1038/s41467-019-12476-z.

10. Maruvada, P., Leone, V., Kaplan, L.M., and Chang, E.B. (2017). The Human Microbiome and Obesity: Moving beyond Associations. Cell Host Microbe *22*, 589–599. https://doi.org/10.1016/j.chom.2017.10.005.

11. Noecker, C., Chiu, H.-C., McNally, C.P., and Borenstein, E. (2019). Defining and Evaluating Microbial Contributions to Metabolite Variation in Microbiome-Metabolome Association Studies. mSystems *4*, e00579-19. https://doi.org/10.1128/mSystems.00579-19.

12. Pearl, J. (2009). Causal inference in statistics: An overview. Statist. Surv. *3*. https://doi.org/10.1214/09-SS057.

13. Schmidt, T.S.B., Raes, J., and Bork, P. (2018). The Human Gut Microbiome: From Association to Modulation. Cell *172*, 1198–1215. https://doi.org/10.1016/j.cell.2018.02.044.

14. Carr, A., Diener, C., Baliga, N.S., and Gibbons, S.M. (2019). Use and abuse of correlation analyses in microbial ecology. ISME J. *13*, 2647–2655. https://doi.org/10.1038/s41396-019-0459-z.

15. Heirendt, L., Arreckx, S., Pfau, T., Mendoza, S.N., Richelle, A., Heinken, A., Haraldsdóttir, H.S., Wachowiak, J., Keating, S.M., Vlasov, V., et al. (2019). Creation and analysis of biochemical constraint-based models using the COBRA Toolbox v.3.0. Nat. Protoc. *14*, 639–702. https://doi.org/10.1038/s41596-018-0098-2.

16. Baldini, F., Heinken, A., Heirendt, L., Magnusdottir, S., Fleming, R.M.T., and Thiele, I. (2019). The Microbiome Modeling Toolbox: from microbial interactions to personalized microbial communities. Bioinformatics *35*, 2332–2334. https://doi.org/10.1093/bioinformatics/bty941.

17. Heinken, A., Ravcheev, D.A., Baldini, F., Heirendt, L., Fleming, R.M.T., and Thiele, I. (2019). Systematic assessment of secondary bile acid metabolism in gut microbes reveals distinct metabolic capabilities in inflammatory bowel disease. Microbiome *7*, 75. https://doi.org/10.1186/s40168-019-0689-3.

18. Baldini, F., Hertel, J., Sandt, E., Thinnes, C.C., Neuberger-Castillo, L., Pavelka, L., Betsou, F., Krüger, R., and Thiele, I.; NCER-PD Consortium (2020). Parkinson's disease-associated alterations of the gut microbiome predict disease-relevant changes in metabolic functions. BMC Biol. *18*, 62. https://doi.org/10.1186/s12915-020-00775-7.

19. Hertel, J., Harms, A.C., Heinken, A., Baldini, F., Thinnes, C.C., Glaab, E., Vasco, D.A., Pietzner, M., Stewart, I.D., Wareham, N.J., et al. (2019). Integrated Analyses of Microbiome and Longitudinal Metabolome Data Reveal Microbial-Host Interactions on Sulfur Metabolism in Parkinson's Disease. Cell Rep. *29*, 1767–1777.e8. https://doi.org/10.1016/j.celrep.2019.10.035.

20. Coyte, K.Z., and Rakoff-Nahoum, S. (2019). Understanding Competition and Cooperation within the Mammalian Gut Microbiome. Curr. Biol. *29*, R538–R544. https://doi.org/10.1016/j.cub.2019.04.017.

21. Francescone, R., Hou, V., and Grivennikov, S.I. (2014). Microbiome, Inflammation, and Cancer. Cancer J. *20*, 181–189. https://doi.org/10.1097/PPO.0000000000000048.

22. Kim, J.S., and de La Serre, C.B. (2018). Diet, gut microbiota composition and feeding behavior. Physiol. Behav. *192*, 177–181. https://doi.org/10.1016/j.physbeh.2018.03.026.

23. Greenwood-Van Meerveld, B., Johnson, A.C., and Grundy, D. (2017). Gastrointestinal Physiology and Function. In Gastrointestinal Pharmacology Handbook of Experimental Pharmacology, B. Greenwood-Van Meerveld, ed. (Springer International Publishing), pp. 1–16. https://doi.org/10.1007/164_2016_118.

24. De Angelis, M., Ferrocino, I., Calabrese, F.M., De Filippis, F., Cavallo, N., Siragusa, S., Rampelli, S., Di Cagno, R., Rantsiou, K., Vannini, L., et al. (2020). Diet influences the functions of the human intestinal microbiome. Sci. Rep. *10*, 4247. https://doi.org/10.1038/s41598-020-61192-y.

25. Heinken, A., and Thiele, I. (2022). Microbiome Modelling Toolbox 2.0: efficient, tractable modelling of microbiome communities. Bioinformatics *38*, 2367–2368. https://doi.org/10.1093/bioinformatics/btac082.

26. Hertel, J., Heinken, A., Martinelli, F., and Thiele, I. (2021). Integration of constraint-based modeling with fecal metabolomics reveals large deleterious effects of Fusobacterium spp. on community butyrate production. Gut Microb. *13*, 1–23. https://doi.org/10.1080/19490976.2021.1915673.

27. Spirtes, P., Clark, G., and Scheines, R. (2001). Causation, Prediction, and Search, Second Edition (MIT Press).

28. Truong, D.T., Franzosa, E.A., Tickle, T.L., Scholz, M., Weingart, G., Pasolli, E., Tett, A., Huttenhower, C., and Segata, N. (2015). MetaPhlAn2 for enhanced metagenomic taxonomic profiling. Nat. Methods *12*, 902–903. https://doi.org/10.1038/nmeth.3589.

29. Heinken, A., and Thiele, I. (2015). Systems biology of host–microbe metabolomics. WIREs Mechanisms of Disease *7*, 195–219. https://doi.org/10.1002/wsbm.1301.

30. Kovatcheva-Datchary, P., Shoaie, S., Lee, S., Wahlström, A., Nookaew, I., Hallen, A., Perkins, R., Nielsen, J., and Bäckhed, F. (2019). Simplified Intestinal Microbiota to Study Microbe-Diet-Host Interactions in a Mouse Model. Cell Rep. *26*, 3772–3783.e6. https://doi.org/10.1016/j.celrep.2019.02.090.

31. Lavin, R., DiBenedetto, N., Yeliseyev, V., Delaney, M., and Bry, L. (2018). Gnotobiotic and Conventional Mouse Systems to Support Microbiota Based Studies. Curr. Protoc. Immunol. *121*, e48. https://doi.org/10.1002/cpim.48.

32. Lukovac, S., Belzer, C., Pellis, L., Keijser, B.J., de Vos, W.M., Montijn, R.C., and Roeselers, G. (2014). Differential Modulation by Akkermansia muciniphila and Faecalibacterium prausnitzii of Host Peripheral Lipid Metabolism and Histone Acetylation in Mouse Gut Organoids. mBio *5*, e01438-14. https://doi.org/10.1128/mBio.01438-14.

33. Magnúsdóttir, S., and Thiele, I. (2018). Modeling metabolism of the human gut microbiome. Curr. Opin. Biotechnol. *51*, 90–96. https://doi.org/10.1016/j.copbio.2017.12.005.

34. Lamichhane, S., Sen, P., Dickens, A.M., Orešič, M., and Bertram, H.C. (2018). Gut metabolome meets microbiome: A methodological perspective to understand the relationship between host and microbe. Methods *149*, 3–12. https://doi.org/10.1016/j.ymeth.2018.04.029.

35. Witte, J., and Didelez, V. (2019). Covariate selection strategies for causal inference: Classification and comparison. Biom. J. *61*, 1270–1289. https://doi.org/10.1002/bimj.201700294.

36. Burgess, S., Small, D.S., and Thompson, S.G. (2017). A review of instrumental variable estimators for Mendelian randomization. Stat. Methods Med. Res. *26*, 2333–2355. https://doi.org/10.1177/0962280215597579.

37. Bonder, M.J., Kurilshikov, A., Tigchelaar, E.F., Mujagic, Z., Imhann, F., Vila, A.V., Deelen, P., Vatanen, T., Schirmer, M., Smeekens, S.P., et al. (2016). The effect of host genetics on the gut microbiome. Nat. Genet. *48*, 1407–1412. https://doi.org/10.1038/ng.3663.

38. Kurilshikov, A., Wijmenga, C., Fu, J., and Zhernakova, A. (2017). Host Genetics and Gut Microbiome: Challenges and Perspectives. Trends Immunol. *38*, 633–647. https://doi.org/10.1016/j.it.2017.06.003.

39. Gudmundsson, S., and Thiele, I. (2010). Computationally efficient flux variability analysis. BMC Bioinf. *11*, 489. https://doi.org/10.1186/1471-2105-11-489.

40. Dawid, A.P. (2010). Beware of the DAG!. In Proceedings of Workshop on Causality: Objectives and Assessment at NIPS 2008, I. Guyon, D. Janzing, and B. Schölkopf, eds. (PMLR)), pp. 59–86.

41. Aalen, O.O., Røysland, K., Gran, J.M., Kouyos, R., and Lange, T. (2016). Can we believe the DAGs? A comment on the relationship between causal DAGs and mechanisms. Stat. Methods Med. Res. *25*, 2294–2314. https://doi.org/10.1177/0962280213520436.

42. Jamshidi, N., and Palsson, B.Ø. (2009). Flux-Concentration Duality in Dynamic Nonequilibrium Biological Networks. Biophys. J. *97*, L11–L13. https://doi.org/10.1016/j.bpj.2009.06.049.

CellPress
OPEN ACCESS

43. Heinken, A., Hertel, J., Acharya, G., Ravcheev, D.A., Nyga, M., Okpala, O.E., Hogan, M., Magnúsdóttir, S., Martinelli, F., Nap, B., et al. (2023). Genome-scale metabolic reconstruction of 7,302 human microorganisms for personalized medicine. Nat. Biotechnol. *41*, 1320–1331. https://doi.org/10.1038/s41587-022-01628-0.

44. Thiele, I., Sahoo, S., Heinken, A., Hertel, J., Heirendt, L., Aurich, M.K., and Fleming, R.M. (2020). Personalized whole-body models integrate metabolism, physiology, and the gut microbiome. Mol. Syst. Biol. *16*, e8982. https://doi.org/10.15252/msb.20198982.

45. Hertel, J., Frenzel, S., König, J., Wittfeld, K., Fuellen, G., Holtfreter, B., Pietzner, M., Friedrich, N., Nauck, M., Völzke, H., et al. (2019). The informative error: A framework for the construction of individualized phenotypes. Stat. Methods Med. Res. *28*, 1427–1438. https://doi.org/10.1177/0962280218759138.

46. Thiele, I., Heinken, A., and Fleming, R.M.T. (2013). A systems biology approach to studying the role of microbes in human health. Curr. Opin. Biotechnol. *24*, 4–12. https://doi.org/10.1016/j.copbio.2012.10.001.

47. Tokudome, S., Imaeda, N., Tokudome, Y., Fujiwara, N., Nagaya, T., Sato, J., Kuriki, K., Ikeda, M., and Maki, S. (2001). Relative validity of a semi-quantitative food frequency questionnaire versus 28 day weighed diet records in Japanese female dietitians. Eur. J. Clin. Nutr. *55*, 735–742. https://doi.org/10.1038/sj.ejcn.1601215.

48. Noronha, A., Modamio, J., Jarosz, Y., Guerard, E., Sompairac, N., Preciat, G., Daníelsdóttir, A.D., Krecke, M., Merten, D., Haraldsdóttir, H.S., et al. (2019). The Virtual Metabolic Human database: integrating human and gut microbiome metabolism with nutrition and disease. Nucleic Acids Res. *47*, D614–D624. https://doi.org/10.1093/nar/gky992.

49. Benjamini, Y. (2010). Discovering the false discovery rate: False Discovery Rate. J. Roy. Stat. Soc. B *72*, 405–416. https://doi.org/10.1111/j.1467-9868.2010.00746.x.

STAR★METHODS

KEY RESOURCES TABLE

REAGENT or RESOURCE	SOURCE	IDENTIFIER
**Deposited data**		
Metagenomic and metabolomic quantifications, meta-data	Yachida et al.[7]	https://static-content.springer.com/esm/art%3A10.1038%2Fs41591-019-0458-7/MediaObjects/41591_2019_458_MOESM3_ESM.xlsx
Mapped Abundances onto AGORA1 (normCoverage_CRC.csv)	THIS PAPER	10.5281/zenodo.8301660
Mapping from keggIDs to AGORA1 identifiers (AGORA1_metabolites_abbr.csv)	THIS PAPER	10.5281/zenodo.8301660
Mapping from keggIDs to metabolite names (keggidname.csv)	THIS PAPER	10.5281/zenodo.8301660
Computed maximum secretion fluxes (inputDiet_net_secretion_fluxes.csv)	THIS PAPER	10.5281/zenodo.8301660
Computed model statistics (ModelStatistics.csv)	THIS PAPER	10.5281/zenodo.8301660
**Software and algorithms**		
MATLAB 2018b	MathWorks Inc.	https://de.mathworks.com
IBM ILOG CPLEX Optimization Studio	International Business Machines Corporation (IBM)	https://www.ibm.com
COBRA Toolbox	Creation and Analysis of Biochemical Constraint-Based Models Using the COBRA Toolbox[15]	https://github.com/opencobra/cobratoolbox
Microbiome Modeling Toolbox	Microbiome Modeling Toolbox 2.0: efficient, tractable modeling of microbiome communities[25]	https://github.com/opencobra/cobratoolbox/tree/master/src/analysis/multiSpecies/microbiomeModelingToolbox
R	The R Project for Statistical Computing	https://www.r-project.org
Stata 16/MP	Stata: Statistical software for data science	https://www.stata.com
R-script performing *in silico in vivo* permutation tests (InSilicoInVivoPatternAnalysis.R)	THIS PAPER	https://doi.org/10.5281/zenodo.8301660

RESOURCE AVAILABILITY

Lead contact

Further information and requests for resources and reagents should be directed to and will be fulfilled by the lead contact, Ines Thiele (ines.thiele@nuigalway.ie).

Materials availability

This study did not generate any new reagents and is based on *in silico* computations relying on publicly available data.

Data and code availability

- This paper analyzes existing, publicly available data. The accession numbers for the datasets are listed in the key resources table.
- The simulation results and all necessary information as well as the original code has been deposited at https://github.com/ThieleLab/Microbiome_Metabolome and is publicly available as of the date of publication. DOIs are listed in the key resources table.
- Any additional information required to reanalyze the data reported in this paper is available from the lead contact upon request.

METHOD DETAILS

Study sample

The study sample consisted of the Japanese colorectal cancer cohort data obtained from Yachida et al.,[7] which included for 347 individuals (220 colorectal cancer cases and 127 healthy controls) shot-gun sequencing data for fecal metagenomics and mass spectrometric metabolome data including quantifications for 450 metabolites. Sequencing reads and taxonomic assignments had been performed using the MetaPhlAn2 pipeline.[28] Furthermore, meta-data on age, sex, and BMI were available and were included in the re-analyses of the data. The details on metagenomic and metabolomic measurements, can be found in the stem publication of the data of Yachida et al.[7] The raw metabolome data and the microbiome abundance data can be found in the supplementary material of the stem paper (https://static-content.springer.com/esm/art%3A10.1038%2Fs41591-019-0458-7/MediaObjects/41591_2019_458_MOESM3_ESM.xlsx).

Construction of sample-specific gut microbiota models

Relative abundances on the species level for the 347 samples were obtained from the supplementary material (https://static-content.springer.com/esm/art%3A10.1038%2Fs41591-019-0458-7/MediaObjects/41591_2019_458_MOESM3_ESM.xlsx) of Yachida et al.[7] In the first step, the quantified species were mapped onto the reference set of 818 microbial metabolic reconstructions (AGORA).[4] Next, COBRA microbial community modeling was done using mgPipe of the Microbiome Modelling Toolbox.[16,25] The input data were relative abundances of the present microbes of each sample and their genome-scale reconstructions obtained from AGORA. To use higher-level taxonomical data, pan-reconstructions were created using the "createPanModels" function of the Microbiome Modeling Toolbox. The function combines the reactions of multiple strains belonging to the same species and creates a combined biomass reaction as the average of all biomass reactions present. A personalized microbiome community model was created by taking the existing (pan-)models of the sample and connecting them through a joint lumen compartment ([u]).[46] In these models, each microbe is able to transport metabolites through a corresponding metabolite exchange reaction from the microbe-specific extracellular space to the lumen compartment. The lumen compartment ([u]) consists of all metabolites that can be transported by at least one microbe in the sample and also connects to a fecal ([fe]) and a diet ([d]) compartment. For each metabolite in the diet and fecal compartment, a sink reaction was added. This setup allows the embedding of diet constraints and the computation of maximum uptake and secretion from/to the environment, respectively. Each community model was parametrized by the biomass reactions of each microbes present $bm_1,\ldots,bm_n$ and the relative microbial abundances $a_{m_1},\ldots,a_{m_n}$ in the respective metagenomic sample to obtain a community biomass reaction $cm = a_{m_1}bm_1 + \ldots + a_{m_n}bm_n$. The community biomass reaction flux was set to be between 0.4 and 1 mmol/person/day, representing fecal excretion of once every three days to daily. Diet constraints were applied by constraining the lower bound of the corresponding sink reaction of the diet compartment and corresponded to an average Japanese Diet as described previously[43] (Table S5) based on the diet data reported in Tokudome et al.[47] and Noronha al.[48] For each existing exchange reaction IEX_{m_i,n_j} of a microbe m_i and a metabolite n_j coupling constraints: $IEX_{m_i,n_j} - 400bm_i \leq 0$ and $IEX_{m_i,n_j} + 400bm_i \geq 0$ were generated. The resulting joint matrix of the individual (pan-)genome-scale reconstructions, connected by the different compartments, the community biomass reaction as well as the coupling constraints equipped with the diet constraints were used to calculate the maximum secretion and uptake capacity.

Simulations

The net community secretion flux values were determined as described in Heinken et al.[17] Briefly, for each metabolite n_j that could be transported by at least one AGORA model included in the microbial community models, flux variability analysis (FVA)[39] was performed for the respective dietary and fecal secretion exchanges by using the community biomass as initial stated objective function:

$$\begin{gathered} \max_{\mathbf{v}} \mathbf{c}^{\mathbf{T}}\mathbf{v}, \\ \text{subject to } \mathbf{S}\mathbf{v} = \mathbf{0}, \\ \mathbf{v_l} \leq \mathbf{v} \leq \mathbf{v_u}. \end{gathered} \quad \text{(Equation 12)}$$

where $\mathbf{S}$ is the mxn stoichiometric matrix with m metabolites and n reactions, $\mathbf{c}^T = (0,\ldots,0,1)$, the vector representing the coefficients of the linear objective function, $\mathbf{v}^T = (v_1,\ldots,v_{n-1},cm)$ the flux vector and $\mathbf{v_l}, \mathbf{v_u}$ represent the upper and lower bounds respectively. Let w be the solution of 12. FVA solved the optimization problems

$$\begin{gathered} \min_{\mathbf{v}} \text{ and } \max_{\mathbf{v}} v_i, \\ \text{subject to } \mathbf{S}\mathbf{v} = \mathbf{0}, \\ cm = w, \\ \mathbf{v_l} \leq \mathbf{v} \leq \mathbf{v_u}. \end{gathered} \quad \text{(Equation 13)}$$

For each metabolite n_j present in the lumen, FVA of the sink reaction of the diet and fecal compartment was performed, while the constraints of the community biomass reaction were set to the value obtained in 12. FVA resulted in values for $minDiet_{n_j}$, $maxDiet_{n_j}$, $minFaecal_{n_j}$, and $maxFaecal_{nj}$. The maximum production $maxProd_{nj}$ and uptake capacity $maxUpt_{nj}$ were calculated as

$$maxProd_{nj} = \left|maxFaecal_{nj} + minDiet_{n_j}\right|, \quad \text{(Equation 14)}$$

and

$$maxUpt_{nj} = \left|minFaecal_{nj} + maxDiet_{n_j}\right|. \quad \text{(Equation 15)}$$

The maximal net secretion flux ($maxProd_{nj}$), which corresponds to the absolute value of the difference between the maximal flux through the fecal secretion exchange reaction and the minimal flux through the corresponding dietary uptake exchange reaction, were calculated subsequently for each metabolite. This has been done for each personalized model. All simulations were performed in MATLAB (Mathworks, Inc.) version R2018b with IBM CPLEX (IBM) as the linear programming solver. The simulations were carried out using the COBRA Toolbox[15] and the Microbiome Modelling Toolbox.[16,25]

QUANTIFICATION AND STATISTICAL ANALYSIS

Statistical operationalization of *in vivo* in silico association pattern analyses

In silico in vivo association pattern analyses consist of three steps. First, the *in vivo* association pattern was determined by calculating the associations between species and metabolite concentration. Second, the *in silico* association pattern between species and community net secretion fluxes was determined. Third, the pattern of *in silico* association was analyzed together with the pattern of *in vivo* associations. The *in silico in vivo* association pattern analyses was performed on all 347 cases with valid COBRA microbial community models. All statistical analysis was performed within the R environment (https://www.r-project.org/). Figure generating partly was performed in R and partly using STATA 16/MP, (StataCorp LLC, College Station, Texas).

Species presence *in vivo* association studies

To generate the *in vivo* association pattern for species presence, we performed linear regressions with the log fecal concentration as response variable, the species presence (binary: present vs. not present) as predictor of interest, while including age, BMI, sex, and study group (binary: colorectal cancer vs. healthy controls) as covariates. To account for potential heteroscedasticity, heteroscedastic robust standard errors were used. This regression model was performed for each microbial species found in at least 10% of the samples, resulting in 148 included species, and for all metabolites having non-zero measurements in at least 50% of the cases, resulting into 54 metabolites included in the analyses. Note that, by using log transformations for the fecal concentrations, we treated zero concentration measures as missing values. We then retrieved the regression coefficient of the species presence variable, which referred in this case to the difference in mean log concentration between the individuals having a certain species in their gut microbiome and those not having this species conditional on the included vector of covariates. To assess significance, FDR correction[49] was applied correcting for $148 * 54 = 7{,}992$ tests. The regression model is given in Equation 16 with $\boldsymbol{Y}_{\boldsymbol{Concentration}_i}$ denoting the fecal concentration of metabolite i, $\boldsymbol{X}_{\boldsymbol{p}\boldsymbol{j}}$ the species presence of species j. The regression coefficient $b_{Cp_{ij}}$ is the coefficient of interest:

$$\log\left(\boldsymbol{Y}_{\boldsymbol{Concentration}_i}\right) = b_{0ij} + b_{Cp_{ij}}\boldsymbol{X}_{\boldsymbol{p}\boldsymbol{j}} + b_{2ij}\boldsymbol{Age} + b_{3ij}\boldsymbol{BMI} + b_{4ij}\boldsymbol{Sex} + b_{5ij}\boldsymbol{Group}. \quad \text{(Equation 16)}$$

Full results can be found in the Table S1.

Species presence *in silico* association studies

To derive the *in silico* species presence association pattern, community net secretion fluxes for the 54 metabolites measured in more than 50% of the cases were calculated as described above. Then, an analogous series of regressions to the *in vivo* species presence metabolite association models were performed exchanging the log fecal concentration with the net community secretion flux values. The net community secretion flux values were not log transformed, because their distributions were not consistently right-skewed as it was the case for the fecal concentrations. Then, the regression coefficients of the microbial species presence variable were extracted. The corresponding regression model is shown in Equation 17 with $\boldsymbol{Y}_{Flux_i}$ denoting the fecal concentration of metabolite i, $\boldsymbol{X}_{\boldsymbol{p}\boldsymbol{j}}$ the species presence of species j. The regression coefficient $b_{Fp_{ij}}$ is the coefficient of interest:

$$\boldsymbol{Y}_{Flux_i} = b_{0ij} + b_{Fp_{ij}}\boldsymbol{X}_{\boldsymbol{p}\boldsymbol{j}} + b_{2ij}\boldsymbol{Age} + b_{3ij}\boldsymbol{BMI} + b_{4ij}\boldsymbol{Sex} + b_{5ij}\boldsymbol{Group}. \quad \text{(Equation 17)}$$

Full results can be found in the supplemental material (Table S3).

Species abundance *in vivo* association studies

To calculate the species abundance *in vivo* association pattern, we formulated analogous models to Equation 17 by exchanging the microbial species presence variable with the standardized species abundance, such that all abundances had the variance of one. This step is necessary as shown in the formalization of the methodology. All other aspects remained the same. Full results can be found in Table S2.

Species abundance *in silico* association studies

The species presence *in silico* association pattern were derived via Equation 18, exchanging the microbial species presence variable for the species abundance. Once, again all other aspects of regression modeling remained the same. Again, full results can be found in Table S4.

In silico in vivo association pattern analyses

We retrieved two pairs of association statistics (species presence pattern and species abundance pattern). To analyze the *in silico in vivo* association pattern, we calculated for each of the 54 metabolites a linear regression with the *in vivo* regression species metabolite regression coefficients as response variable and the corresponding *in silico* regression coefficient as predictor using once again heteroscedastic robust standard errors. The utilized regression equation is displayed in 3 and the corresponding slope β_{1i} was then tested on being zero. Note that $\boldsymbol{b_{Fi}}$ and $\boldsymbol{b_{Ci}}$ are now vectors of regression coefficients, originating from the *in silico* and *in vivo* association studies of the metabolite *i:*

$$\boldsymbol{b_{Ci}} = \beta_{0i} + \beta_{1i}\boldsymbol{b_{Fi}}. \quad \text{(Equation 18)}$$

Because the metabolite concentrations as well as the microbiome abundances are intercorrelated, the estimated coefficients of the regression model (3) are not independent. The estimated coefficients of Equation 18 do not follow a normal distribution and therefore the transformed R^2:$F = \frac{R^2}{1-R^2}(n - 2)$ under $H_0: \beta_{1i} = 0$ is not $F(1, n - 2)$ distributed (Figure S1). This property can result in too liberal p values and false rejections (type I errors) of H_0. We resolved this issue by shuffling the computed *in silico* fluxes 5,000 times and calculating the corresponding measures for each permutation and then calculating the probability of how many computed permutations of the empirical distribution resulted in a measure greater than the one with the original flux vector. Significance of the regression model (Equation 18) was determined with the standard procedure ($p < 0.05$) and the method based on permutations of the flux vector, resulting in testing 54 metabolites.

In a second step, we analyzed the agreement in sign of the *in silico* and *in vivo* association statistics. To this end, the sign of the *in vivo* association statistics was tabulated against the sign of the *in silico* association statistics per metabolite. Once again, significance was assessed using the same methodology of the permutated flux vectors as done for determining the significance of β_{1i}. Note that the statistical power to detect significant sign agreement depends on the base rate of positive, respectively negative, associations. Statistical power will be low if nearly all associations are either positive or negative.

For comparison and reference, Table S3 and S4 also include p values derived from classical hypothesis testing, showing as expected more liberal p values. For interpretation of results, only the permutation-based p values were utilized.

Cell Reports Methods, Volume *3*

Supplemental information

Causal inference on microbiome-metabolome relations in observational host-microbiome data via *in silico in vivo* association pattern analyses

Johannes Hertel, Almut Heinken, Daniel Fässler, and Ines Thiele

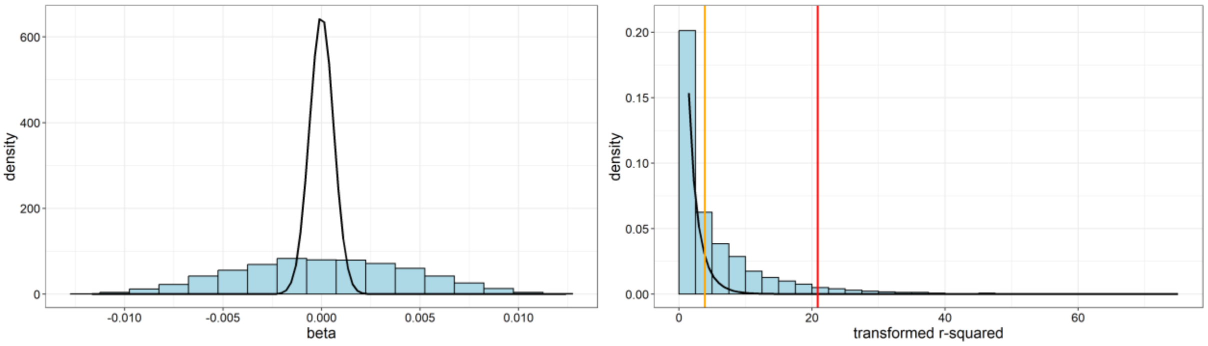

Figure S1. Histogram of estimated regression coefficients (beta) characterising *in silico in vivo* association pattern from 5,000 permutations, related to STAR Methods. Histogram of estimated regression coefficients (beta) characterising *in silico in vivo* association pattern from 5,000 permutations of butyrate net secretion fluxes and the expected density of the normal distribution (left panel). On the right panel the distribution of the corresponding transformed R^2 values, density function of the $\mathrm{F}(1{,}146)$ distribution (black), the 5% quantile (orange line) and the empirical 5% quantile of the transformed R^2 (red line) are shown. Empirical distributions indicate that nominal p-values will be too liberal.

Note S1: Formalisation of metabolic host-microbiome systems via structural equation modelling, related to Figure 2

As noted in the main body of the manuscript, we define:

a) $Microbiome\ community\ composition\ \boldsymbol{a} := (a_1, a_{2,}, \dots, a_L) \in [0,1]^L\ with\ \sum_{l=1}^{L} a_l = 1$
b) $Host\ characterisation\ \boldsymbol{h} := (h_1, h_{2,}, \dots, h_K) \in \mathbb{R}^K$
c) $Community\ net\ secretion\ \boldsymbol{f} := (f_1, f_{2,}, \dots, f_J) \in \mathbb{R}^J\ of\ J\ metabolite\ fluxes$
d) $Host\ metabolome\ \boldsymbol{c} := (c_1, c_{2,}, \dots, c_J) \in \mathbb{R}^J\ of\ J\ metabolite\ concentrations$

We will call the vector $(\boldsymbol{a}, \boldsymbol{h}, \boldsymbol{f}, \boldsymbol{c})$ metabolic microbiome-host-system, dealing with *N* independent and identically distributed (IID) realisations of the vector $(\boldsymbol{a}, \boldsymbol{h}, \boldsymbol{c})_{\boldsymbol{1}}, (\boldsymbol{a}, \boldsymbol{h}, \boldsymbol{c})_{\boldsymbol{2}}, \dots, (\boldsymbol{a}, \boldsymbol{h}, \boldsymbol{c})_{\boldsymbol{N}}$, with $\boldsymbol{f_n}$ being approximated by a deterministic known function based on microbiome composition $\boldsymbol{a_n}$, the genome-scale reconstructions of the microbes detected in $\boldsymbol{a_n}$ and constant diet constraints across the whole population of metabolic microbiome-host-systems. Notably, we do not need to apply distributional assumptions other than the metabolic microbiome-host-systems being IID. We will generally assume that (a, h, f, c) are nontrivial with finite variances. The whole methodology is therefore nonparametric.

We assume that the interrelations can be reasonably approximated by the following structural linear equation system:

(1) $a_l = u_l + \sum_{k=1}^{K} b_{ah_{lk}} h_k$, leading to $\boldsymbol{a} = \mathbf{B_{ah}} \boldsymbol{h} + \boldsymbol{u}\ with\ \mathbf{B_{ah}} \in MAT(L, K)\ and\ \boldsymbol{u} = (u_1, u_{2,}, \dots, u_L)$,

(2) $f_j = \sum_{l=1}^{L} b_{fa_{jl}} a_l$, leading to $\boldsymbol{f} = \mathbf{B_{fa}} \boldsymbol{a}\ with\ \mathbf{B_{fa}} \in MAT(J, L)$,

(3) $c_j = \sum_{i=1}^{J} b_{cf_{ji}} f_i + \sum_{k=1}^{K} b_{ch_{jk}} h_k + \varepsilon_j, leading\ to\ \boldsymbol{c} = \mathbf{B_{cf}} \boldsymbol{f} + \mathbf{B_{ch}} \boldsymbol{h} + \boldsymbol{\varepsilon}\ with\ \mathbf{B_{cf}} \in MAT(J, J),\ \mathbf{B_{ch}} \in MAT(J, K),\ \boldsymbol{\varepsilon} = (\varepsilon_1, \varepsilon_{2,}, \dots, \varepsilon_J)$.

As described in the main text, we assume:
i) $\boldsymbol{u} \perp\!\!\!\perp \boldsymbol{h}, (stochastic\ independence\ between\ \boldsymbol{u}\ and\ \boldsymbol{h})$
ii) $\boldsymbol{\varepsilon} \perp\!\!\!\perp \boldsymbol{h}, \boldsymbol{f}\ (stochastic\ independence\ between\ \boldsymbol{\varepsilon}\ and\ \boldsymbol{h}, \boldsymbol{f})$
iii) $\mathbf{B_{fa}} \mathbf{B_{ah}} = \mathbf{0}\ (approximate\ orthogonality\ of\ \mathbf{B_{ah}}\ and\ \mathbf{B_{fa}})$

In this supplementary note, we will derive three attributes of the above constructed system of structural equations.

We define furthermore as in the main document:
(4) $\hat{b}_{fa_{jl}} = \frac{COV(f_j, a_l)}{VAR(a_l)}$, $\hat{\boldsymbol{b}}_{\boldsymbol{fa}_{j.}} = (\hat{b}_{fa_{j1}}, \hat{b}_{fa_{j2}}, \dots, \hat{b}_{fa_{jL}})$ *(in silico association statistics)*
which is the vector of regression coefficients of the regression prediction score:
(5) $\hat{f}_j = \hat{b}_{fa_{jl}} a_l + b_{fa_0}$.
Second, we define
(6) $\hat{b}_{ca_{jl}} = \frac{COV(c_j, a_l)}{VAR(a_l)}$, $\hat{\boldsymbol{b}}_{\boldsymbol{ca}_{j.}} = (\hat{b}_{ca_{j1}}, \hat{b}_{ca_{j2}}, \dots, \hat{b}_{ca_{jL}})$ *(in vivo association statistics)*
which is vector of the regression coefficients of the regressions prediction score:
(7) $\hat{c}_j = \hat{b}_{ca_{jl}} a_l + b_{ca_0}$.

Attribute 1:

The covariance $COV\left(\boldsymbol{b}_{\boldsymbol{fa}_{j.}}, \boldsymbol{b}_{\boldsymbol{ah}_{.k}}\right)$ between the vectors of model parameters $\boldsymbol{b}_{\boldsymbol{fa}_{j.}} = (b_{fa_{j1}}, b_{fa_{j2}}, \dots, b_{fa_{jL}})$ and $\boldsymbol{b}_{\boldsymbol{ah}_{.k}} = (b_{ah_{1k}}, b_{ah_{2k}}, \dots, b_{ah_{Lk}})$ is zero if $Var(\sum_{k=1}^{K} b_k h_k) \neq 0$ for all nontrivial coefficient vectors with $||(b_1, b_2, \dots, b_K)|| \neq 0$.

Proof:

From iii) and ii), it follows that $\sum_{l=1}^{L} b_{ah_{lk}} = 0$ for all $k \in \{1,2,\dots,K\}$, since $\sum_{l=1}^{L} a_l = 1$
(8) $1 = \sum_{l=1}^{L} a_l = \sum_{l=1}^{L} (u_l + \sum_{k=1}^{K} b_{ah_{lk}} h_k) = \sum_{l=1}^{L} u_l + \sum_{l=1}^{L} \sum_{k=1}^{K} b_{ah_{lk}} h_k = \sum_{l=1}^{L} u_l + \sum_{k=1}^{K} h_k \sum_{l=1}^{L} b_{ah_{lk}}$.

As $\boldsymbol{u} \perp\!\!\!\perp \boldsymbol{h}$:

(9) $0 = COV\left(\sum_{l=1}^{L} u_l, \sum_{j=1}^{J} h_j \sum_{l=1}^{L} b_{ah_{lj}}\right) = COV\left(\sum_{l=1}^{L} u_l, \sum_{j=1}^{J} h_j \sum_{l=1}^{L} b_{ah_{lj}}\right) = COV\left(1 - \sum_{l=1}^{L}\sum_{k=1}^{K} b_{ah_{lk}} h_k, \sum_{j=1}^{J} h_j \sum_{l=1}^{L} b_{ah_{lj}}\right) = -COV\left(\sum_{k=1}^{K} h_k \sum_{l=1}^{L} b_{ah_{lk}}, \sum_{j=1}^{J} h_j \sum_{l=1}^{L} b_{ah_{lj}}\right) = -Var\left(\sum_{k=1}^{K} h_k \sum_{l=1}^{L} b_{ah_{lk}}\right)$.

Thus, $\sum_{l=1}^{L} b_{ah_{lk}} = 0$ for all $k \in \{1,2,\ldots,K\}$. Rewriting $COV\left(\boldsymbol{b_{fa_{j\cdot}}}, \boldsymbol{b_{ah_{\cdot k}}}\right)$ as difference between arithmetic means, we get:

(10) $COV\left(\boldsymbol{b_{fa_{j\cdot}}}, \boldsymbol{b_{ah_{\cdot k}}}\right) = Mean\left(\boldsymbol{b_{fa_{j\cdot}}} \boldsymbol{b_{ah_{\cdot k}}}\right) - Mean\left(\boldsymbol{b_{ah_{\cdot k}}}\right) Mean\left(\boldsymbol{b_{fa_{j\cdot}}}\right) = \frac{1}{L}\sum_{i=1}^{L} b_{fa_{ji}} b_{ah_{ik}} - \frac{1}{L}\sum_{i=1}^{L} b_{fa_{ji}} \frac{1}{L}\sum_{i=1}^{L} b_{ah_{ik}} = \frac{1}{L}\sum_{i=1}^{L} b_{fa_{ji}} b_{ah_{ik}} = 0, since\ \mathbf{B_{fa}B_{ah}} = \mathbf{0}.$

□

Attribute 2:

The estimate $\hat{b}_{fa_{jl}}$ is a function of $\boldsymbol{u}$ and $\mathbf{B_{fa}}$ with $\hat{b}_{fa_{jl}} = \frac{COV\left(\sum_{i=1}^{L} b_{fa_{ji}} u_i, u_l\right)}{Var(a_l)}$.

Proof:

We get utilising that $\mathbf{B_{fa}B_{ah}} = \mathbf{0}$ and that $\boldsymbol{u} \perp\!\!\!\perp \boldsymbol{h}$:

(8) $VAR(a_l)\hat{b}_{fa_{jl}} = COV(f_j, a_l) = COV\left(\sum_{i=1}^{L} b_{fa_{ji}} a_i, a_l\right) = COV\left(\sum_{i=1}^{L} b_{fa_{ji}} \left(u_i + \sum_{k=1}^{K} b_{ah_{ik}} h_k\right), a_l\right) = COV\left(\sum_{i=1}^{L} b_{fa_{ji}} u_i + \sum_{i=1}^{L} b_{fa_{ji}} \sum_{k=1}^{K} b_{ah_{ik}} h_k, a_l\right) = COV\left(\sum_{i=1}^{L} b_{fa_{ji}} u_i, a_l\right) + COV\left(\sum_{i=1}^{L} b_{fa_{ji}} \sum_{k=1}^{K} b_{ah_{ik}} h_k, a_l\right) = COV\left(\sum_{i=1}^{L} b_{fa_{ji}} u_i, a_l\right) + COV\left(\sum_{k=1}^{K} h_k \sum_{i=1}^{L} b_{fa_{ji}} b_{ah_{ik}}, a_l\right) = COV\left(\sum_{i=1}^{L} b_{fa_{ji}} u_i, a_l\right) = COV\left(\sum_{i=1}^{L} b_{fa_{ji}} u_i, u_l + \sum_{k=1}^{K} b_{ah_{ik}} h_k\right) = COV\left(\sum_{i=1}^{L} b_{fa_{ji}} u_i, u_l\right)$.

Thus, the estimate $VAR(a_l)\hat{b}_{fa_{jl}}$ is only dependent on $\boldsymbol{u}$ (microbiome intrinsic) and $\mathbf{B_{fa}}$.

□

Attribute 3:

The estimate $\hat{b}_{ca_{jl}}$ can be written as $\hat{b}_{ca_{jl}} = [b_{cf_{jj}} \hat{b}_{fa_{jl}} + COV(\sum_{k=1}^{K} b_{ch_{jk}} h_k, \sum_{t=1}^{K} b_{ah_{lt}} h_t)] \frac{1}{VAR(a_l)}$.

Proof:

Using the structural equations (1)-(3), we derive:

(9) $VAR(a_l)\hat{b}_{ca_{jl}} = COV(c_j, a_l) = COV\left(b_{cf_{jj}} f_j + \sum_{k=1}^{K} b_{ch_{jk}} h_k + \varepsilon_j, a_l\right) = b_{cf_{jj}} COV(f_j, a_l) + COV(\sum_{k=1}^{K} b_{ch_{jk}} h_k, a_l) = b_{cf_{jj}} \hat{b}_{fa_{lj}} + COV(\sum_{k=1}^{K} b_{ch_{jk}} h_k, u_l + \sum_{t=1}^{T} b_{ah_{lt}} h_t) = b_{cf_{jj}} \hat{b}_{fa_{lj}} + COV(\sum_{k=1}^{K} b_{ch_{jk}} h_k, \sum_{t=1}^{T} b_{ah_{lt}} h_t)$.

□
